# Investigating Ionic Effects Applied to Water Based Organocatalysed Aldol Reactions

**DOI:** 10.3390/ijms12129083

**Published:** 2011-12-07

**Authors:** Joshua P. Delaney, Luke C. Henderson

**Affiliations:** Chemistry and Systems Biology, Strategic Research Center for Biotechnology, Deakin University, Waurn Ponds Campus, Geelong, Victoria 3216, Australia; E-Mail: jpdel@deakin.edu.au

**Keywords:** ionic solution, organocatalysis, aldol, water

## Abstract

Saturated aqueous solutions of various common salts were examined for their effect on aqueous aldol reactions catalysted by a highly active C_2_-symmetric diprolinamide organocatalyst developed in our laboratory. With respect to the aldol reaction between cyclohexanone and 4-nitrobenzaldehyde, deionised water was always a superior medium to salt solutions though some correlation to increasing anion size and depression in enantiomeric excess could be observed. Additionally, the complete inhibition of catalyst activity observed when employing tap water could be alleviated by the inclusion of ethylenediaminetetraacetate (EDTA) into the aqueous media prior to reaction initiation. Extension of these reaction conditions demonstrated that these ionic effects vary on a case-to-case basis depending on the ketone/aldehyde combination.

## 1. Introduction

The field of organocatalysis has undergone a dramatic increase in interest since the seminal work of List and Barbas demonstrated the great potential and broad scope of organocatalysed reactions [1–3]. As the benefits of organocatalysts are realized, such as their benign nature toward air and moisture, the introduction of asymmetric carbon centers via proline based catalysis is now becoming a viable tool to the synthetic organic chemist. Organocatalysed reactions carried out in the presence of water have seen increasing popularity in recent years as the focus of much research has shifted toward the implementation of greener principles and more environmentally friendly practices. Because of this “Green” approach, the organocatalysis of reactions “on-water” or “in-water” emerged at the forefront of catalysis research [4–20]. Recently a variety of groups have reported the potential benefits water (or brine solutions) can have within these systems, demonstrating not just water tolerant organocatalysts, but catalysts that benefit from interactions afforded by an aqueous environment [21]. It’s often observed that once a catalyst has been tailored toward compatibility with aqueous environments, water itself then encourages further activity and enantiomeric induction through hydrogen bonding networks participating in theorized transition states. Despite this, it is not uncommon for organocatalysis carried out in aqueous environments to require a polar organic co-solvent (e.g., DMSO or DMF) and/or a rate enhancing additive such as benzoic acid, trifluoroacetic acid among others to compensate for a deficiency in water compatibility [22,23].

The most often investigated aqueous solvent system is MilliQ or deionized water, a system employed by groups such as Hayashi *et al*. whereby *trans-*4-TBDPSO proline facilitated one of the first aldol reactions in an aqueous media, free from an organic co-solvent [24]. The aldol products of this study showed a near perfect enantiopurity and very high catalytic activity (78% yield in 48 h with organocatalyst loading of 1 mol%). Similar high activities and enantiopurities were achieved by Mase *et al.* thought the use of long chain l-proline derivatives in an aqueous environment through the introduction of surfactants [25] and organic acids [26].

Often when investigating aqueous environments for organocatalyst optimization, brine is employed as an alternative to deionised or milliQ water in order to further probe the tolerance of a catalyst toward complex aqueous environments. In most cases the ionic solution is seen to have an effect on the reaction rate and enantiopurity of products obtained, as described by Wang *et al.*, though this change is not always an improvement [27].

The aldol reaction is one of the most fundamental C–C bond forming tools available to an organic chemist [28,29], and over the past decade has established itself as the benchmark reaction for organocatalysis investigation. Despite the impact afforded by ions in solution, the investigation into ionic solutions has, by and large, been restricted to saturated solutions of sodium chloride and/or tap water. In light of this lack of in depth investigation, this study endeavors initially to evaluate the effects of common ionic solutions have on an organocatalysed aldol reaction between *p-*nitrobenzaldehyde and cyclohexanone, then expand this study to other ketone/aldehyde variations.

## 2. Results and Discussion

To assess the effect of saturated aqueous ionic solutions on the direct aldol reaction we employed an organocatalyst previously synthesized in our laboratory that has shown to be highly active catalyst for the direct aldol reaction in water [30]. This was considered pertinent as our experience with this catalyst provided a level ground upon which ionic effects could be compared.

### 2.1. Synthesis of Diprolinamide Organocatalyst

**Scheme 1:**
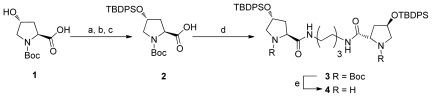
(**a**) NEt_3_, CH_2_Cl_2_, room temperature, BnBr, 67%; (**b**) TBDPS-Cl, Imidazole, DMAP, DMF; (**c**) H_2_, Pd/C, MeOH, 72% for two steps; (**d**) EDCI, HOBt, 1,6-diaminohexane (0.5 equivalent), CH_2_Cl_2_, 80%; (**e**) 10% TFA/CH_2_Cl_2_, 16 h, 99%.

The synthesis of the organocatalyst chosen for this study has been synthesized and investigated in previous studies by our research group, but is provided here for clarity (as shown in Scheme 1) [30]. This synthesis involved the protection of the carboxylate of *trans-*4-OH-*N*-Boc-proline unit **1** with a benzyl ester. The resulting protected species was then sylilated using *tert-*butyldiphenylsilyl chloride. The benzyl group was then removed by catalytic hydrogenolysis resulting in the proline monomer **2** in good yield over three steps.

To synthesize the highly active diprolinamide **4**, *N*-protected proline **2** was coupled to either end of 1,6-diaminohexane via EDCI mediated peptide coupling giving protected dimer **3**. This was followed by quantitative Boc deprotection using 10% TFA in dichloromethane to give the desired organocatalyst **4**. For full synthetic procedure and corresponding spectra of **4** and other synthetic intermediates, please refer to the supplementary information.

### 2.2. Evaluation of Ionic Solution Effects

**Scheme 2:**
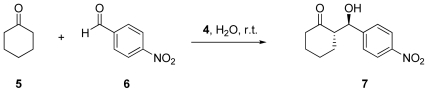
Aldol reaction catalysed by **4**.

With the successful synthesis of diprolinamide **4** completed, a series of organocatalysed aldol reactions were carried out. The aldol reaction of cyclohexanone **5** and *p*-nitrobenzaldehyde **6** was selected as the model system for the comparative study due to the frequency by which this system is used in literature, and due to the extensive research carried out on this system using catalyst **4** established in previous studies (as shown in Scheme 2) [30].

Investigation into the effects of ionic solutions was initiated with an aldol reaction in deionized water to determine a base level of activity (entry 1, Table 1). This reaction was then repeated with the aqueous phase the substituted for tap water (entry 2, Table 1) and brine (entry 4, Table 1) as the aqueous media. These entries represent two of the most widely available solvent systems, and two of the most often reported “pure” water alternatives. Both examples showed significant reduction in catalytic activity when compared to deionized water, tap water in particular displaying a drastic reduction in yield from 91% to 7% (entry 2, Table 1). In both cases a comparable sacrifice in diastereomeric purity was observed. As saturated sodium chloride was the first solution implemented, sodium was chosen as a starting cation, and several anions were investigated.

Examining the application of various sodium salt solutions has afforded mixed results, with sodium fluoride, bromide and iodide (entries 3, 5 and 6, Table 1) decreasing the yield of the desired product (49%, 30% and 17% respectively) when compared to sodium chloride (54%). In parallel to this decrease in yield was a reduction in enantiomeric excess, where a substantial decrease in ee was observed (67%, 43% and 34%) when employing sodium fluoride, bromide and iodide respectively (entries 3, 5 and 6, Table 1). It is worth noting here that in the interest of thoroughness sodium fluoride and potassium fluoride aqueous solutions were used (entries 3 and 8, Table 1) though the inclusion of the fluoride anion in the reaction mixture may be removing the diphenyl-*t*-butylsilyl group from organocatalyst **4**, thus altering the catalyst *in situ*. Sodium acetate (entry 7, Table 1) was also examined as the anion in this case is mildly basic and is a trigonal anion rather than the spherical halide anions discussed above. In this case a higher yield of the aldol product was obtained but it possessed a severely depressed of enantiopurity (38% ee) while diastetreomeric ratio was largely unchanged (81/19) from other salt solutions. Both of these observations may be due to the basic nature of the acetate anion leading to enhanced reaction rate, causing higher yield, in addition to non-specific base catalysed aldol reaction, giving poorly enantio-enriched products. Despite the range of activity allowed by each Na^+^ solution, a uniform diastereomeric ratio was observed, with each product exhibiting a diastereomeric ratio of approximately 80:20.

Shifting focus to the same series of potassium salt solutions, in all cases the reaction performed poorly. Employing potassium fluoride (entry 8, Table 1) gave a very poor yield (23%) while enantiopurity remained moderate (79/22, *anti*/*syn*, 67% ee). It is worth noting that the enantiopurity of compounds obtained in this reaction are very similar to the instance where sodium fluoride was used, thus supporting the notion of *in situ* deprotection. Interestingly the potassium ion solutions showed a conflicting trend with respect to yield when compared to the corresponding sodium solutions. In the case of potassium salts, the yields decreased when chloride (27%) and iodide (32%) were employed respectively (entries 9 and 11, Table 1) while the use of sodium bromide gave a slightly higher yield (44%). It also seems as though the presence of potassium in solution is decreasing overall catalyst activity. When examining the optical purities of these examples there is a similar trend to the sodium series whereby ee decreases when the chloride (45% ee), bromide (35% ee) and iodide (21% ee) anions were employed respectively (entries 9–11, Table 1).

Finally, two groups ((II) metal containing ionic solutions) were investigated, MgCl_2_·7H_2_O and CaCl_2_ (entries 12 and 13, Table 1, respectively). When MgCl_2_·7H_2_O ionic solution is used as a solvent medium for the aldol reaction catalysed by **4**, a reduction in organocatalyst activity (51%) and enantio-induction was observed (55% ee) while dr remained at 80/20 *anti*/*syn*. Interestingly, employing CaCl_2_ (entry 13, Table 1) in the aqueous reaction media gave complete conversion to the desired aldol product (>99%), demonstrating an increased yield compared to that of all prior entries, though the increase in yield for the obtained product was not without its drawbacks as it was isolated as both diastereomerically and enantiomerically racemic (45/55 *anti*/*syn*, 2% ee). The solubilisation of calcium chloride will cause a drop in aqueous pH, which can also facilitate the aldol reaction. In this case, the absence of any chiral catalyst or ligand will promote the formation of racemic products.

It is important to note that these reactions are carried out in an emulsion of the donor ketone within the aqueous phase. Thus it is unusual that the aqueous contents can influence the processes occurring within the hydrophobic pocket in which the catalysed reaction is taking place. A catalysis reaction which is occurring within an aqueous emulsion has dramatic ramifications when considering the reaction mechanism/transition state. In 2007 Jung and Marcus [32] proposed that at the oil-and-water interface there exists free OH groups which protrude into the organic phase (*i.e.*, droplets of the donor ketone). This effect is associated with the highly ordered network of water molecules which surround the oil droplet [33,34]. As such we propose that the means by which the ions in solution are affecting the reaction outcomemay be due to the disruption of the highly ordered nature of the H-bonding network present in water; which may have follow on effects when considering the oil-in-water interface. For example there may not be as many OH groups present to stabilize the highly polar transition state leading to reduced yields and massive reductions in enantiopurity for the aldol products obtained.

Given the dramatic decrease in reaction progression when tap water was employed in place of deionised water (entry 2, Table 1) we reasoned that transition metals which commonly accumulate within tap water may be responsible for this pronounced effect. Due to the extensive copper piping used to plumb water, our initial investigation examined copper salts employing both copper(I) and copper(II) oxidation states (entries 1 and 2, Table 2). The presence of copper cations in both oxidations states had a drastic suppressive effect on reaction progression, giving effectively no product in either case. Due to the minimal conversion of reagents to aldol product **7**, a reliable value for both dr and ee could not be determined. This inhibitory effect of copper has been observed in other studies by Penhoat *et al*. [35]. Interestingly the use of iron(III) chloride (entry 3, Table 2) promoted complete conversion to the desired product but had profound negative influence on the stereochemical purity of the desired compound. The preferred diastereomeric isomer of **7** in this case was *syn* though only by a small margin (42/58, *anti*/*syn*) while no enantiomeric isomer preference was observed (0% ee). The high reaction conversion to the desired aldol products in this case may have arisen due to the decrease in pH of the aqueous environment due to dissolution of the iron(III) chloride. In a similar manner to calcium chloride, the acidic pH may be resulting in non-specific acid catalysed enol formation leading to racemic products.

In a similar vein to the use of copper(I) and (II) salts, the incorporation of nickel(II) chloride into the aqueous reaction media demonstrated almost complete inhibition of catalyst activity (3%, entry 4, Table 2). In this case a diastereomeric ratio was able to be obtained and was consistent with many other examples in this study, remaining at 80/20 *anti*/*syn*. Interestingly, despite the severe depression in reaction progress, the enantiomeric purity of the trace compound isolated was still very high (83% ee). To further investigate the impact of the acetate anion previously discussed (entry 7, Table 1), saturated Zn(OAc)_2_ was selected and implemented as the solvent system (entry 5, Table 2). This ionic system imparted a similar high conversion as that obtained from sodium acetate, though the enantiomeric excess (92% ee) was far superior. Recently, Andreu and Asensio [36] reported the beneficial effects of Zn^2+^ being present in their prolinamide organocatalysed aldol reactions. That study proposed the formation of a Zn-proline complex, which enhanced both the enantioselectivity of the catalyst and decreased the basic nature of the proline nitrogen resulting in non-specific base catalysed reactions. Additionally the presence of Zn(OAc)_2_ has improved the yield and enantiopurity of aldol products of polymer immobilised organocatalysts [36]. Further to this, it should be noted that the solubilisation of transition metal compounds to the aqueous phase can inhibit the amount of organic compounds soluble in the reaction media. As such this may be a contributing factor to the observed results.

One theory considered by the authors to rationalize the suppression of activity within a tap water medium was the potential presence of copper(I) and copper(II) ions in solution. This cation is commonly observed to form complexes in several l-proline or diamino systems presented in literature [38–40].

Having consistently observed the inhibitory effect imparted by several metal ions, it was proposed that the presence of these ions were responsible for the lack of activity observed in the organocatalysed aldol reaction in the presence of tap water (Table 1, entry 2). Under this assumption, we endeavored to rectify this inhibitory effect through the implementation of ethylenediaminetetraacetate (EDTA), a commonly used metal ion chelating agent. To investigate the effect EDTA imparted on the organocatalysed aldol reaction, a solution of tap water containing 5% w/w EDTA was prepared and employed as the solvent. A control experiment to determine the direct effect of EDTA on the aldol reaction was carried out (entry 1, Table 3), resulted in no observed aldol product formation, thus allowing all reaction progress to be attributed to diprolinamide **4**. Repeating this reaction with the organocatalyst demonstrated a near quantitative conversion to the desired product (98%) in 24 h with a diastereomeric ratio of 84:16 (*anti*/*syn)*, and promoted a moderate enatiomeric excess (51%). Interestingly, the activity observed in the presence of EDTA outperformed the reaction carried out in deionised water, though the enantiopurity was significantly sacrificed (51%). This result may arise via a similar effect as observed with sodium acetate (entry 7, Table 1) where the basic acetate groups of EDTA are promoting non-specific aldol reactions, thus lowering enantiomeric excess but enhancing reaction conversion.

In light of these results we were interested in determining if the results obtained by the aldol reaction between cyclohexanone **5** and 4-nitrobenzaldehyde **6** were representative for other benzaldehydes and cyclic ketones–specifically cyclopentanone. Note that the use of the fluoride anion was not investigatied as this served as a potential means to deprotect the *tert*-butyldiphenylsilylether at the 4-position of the prolines present in the organocatalyst **4**. As such this would change the catalyst *in situ* and so would provide misleading information as mentioned earlier. Initially we began by examining benzaldehyde **8** paired with cyclohexanone **5**. In deionized water (entry 1, Table 4) the conversion was only moderate which was attributed to the lack of electron withdrawing group on the aromatic ring (when compared to 4-nitrobenzaldehyde). Nevertheless, the dr remained high (92/8, *anti*/*syn*) and enantiomeric excess was moderate (68%). Unusually, when employing tap water as the reaction medium (entry 2, Table 4) we observed an increase in reaction conversion which is in stark contrast to the observation made with 4-nitrobenzaldehyde (entry 1, Table 2). Though similarly to previous observations the dr decreased slightly (84/16, *anti*/*syn*) and ee was decreased substantially (28%). The presence of EDTA (entry 3, Table 4) corresponded to a marked increase in reaction progression (98%) while enantiopurity of the aldol products obtained were not substantially different to the previous entry (dr 83/17, *anti*/*syn*, 25% ee).

In the case of benzaldehyde **6** Zn(OAc)_2_ (entry 4, Table 4) was observed to have a positive effect on reaction outcome, when applied to this system both the reaction conversion and enantiopurity of the aldol product were severely depressed. The reaction conversion (44%) was similar to that obtained when deionized water was used as the reaction medium though the product dr was mildly reduced (76/24, *anti*/*syn*) while all enantioselectivity seemed to have been removed from this process, as the product was almost racemic (4% ee).

Employing 4-fluorobenzaldehyde as the aldol reaction partner gave very poor reaction conversion when using deionized water as the reaction solvent (21%, entry 5, Table 4). Despite this low conversion, the product obtained possessed very high enantiopurity (dr 91/9, *anti*/*syn*, 92% ee). In a similar result to that of benzaldehyde, the use of tap water gave a substantially higher amount of aldol product (80%, entry 7, Table 4) but in this case the enantiopurity of **9b** was not as severely impeded (dr 86/14, *anti*/*syn*, 68% ee). The incorporation of EDTA into the tap water reaction media also gave a high yield (70%) while leaving dr negligibly changed (85/15, *anti*/*syn*) with a moderate increase to enaniopurity (88% ee). The effect of Zn(OAc)_2_ (entry 8, Table 4) was not as pronounced in this case as the previous, the addition of this compound to the aqueous phase gave a good conversion (70%) and only slightly diminished dr (76/24, *anti*/*syn*) and ee (64%). Indeed these results were very similar to those obtained in this system when tap water was employed (entry 6, Table 4).

Thus far all aldol reactions have been carried out using cyclohexanone **5** as the donor ketone with various aryl aldehydes. To further probe the effect of ionic salt solutions, we broadened our scope to include another cyclic ketone (cyclopentanone) in order to determine if the unpredictable catalyst activities were dependent on both aldol reaction partners. Unfortunately, in all cases this reaction performed poorly with respect to enantiopurity of aldol products obtained, though the opposite (*syn*) diastereomer was obtained in each case. Interestingly deionized water and tap water (entries 9 and 10, Table 4) gave similar reaction conversion while tap water gave products of higher enantiopurity (22%) than deionised water (14%). The addition of EDTA (entry 11, Table 4) improved reaction conversion but had no effect on enantiomeric excess. Finally, Zn(OAc)_2_ facilitated a near quantitative conversion (97%, entry 12, Table 4) of the desired aldol reaction but with little effective chiral induction (14% ee).

## 3. Conclusion

In conclusion we have shown that the use of saturated ionic solutions as reaction media for organocatalysed aldol reactions can have dramatic effect on both the yield and enantiopurity of reaction products. Deionised water always proved superior to aqueous salt solutions with tap water causing an almost complete suppression of catalyst activity. Investigating a range of transition metal solution as reaction media suggested that the presence of copper(I) and/or copper(II) cations in solution were responsible for catalyst inactivation. This action was presumably facilitated by complexation of the diprolinamide catalyst via Cu–N dative bond formation. In light of this, the suppressive action of tap water was then alleviated by the addition of EDTA to the tap water reaction media, prior to the addition of the aldol reagents and organocatalyst. This returned the reaction progression to its full potential and increased the enantiopurity of the aldol product obtained. These ionic effects are not universal and seem to vary on a case-to-case basis depending on both reaction partners in the aldol reaction, again this may arise due to effects at the oil-in-water interface with respect to hydrogen bonding and reaction volume. To elucidate a rationale for these effects, a wider scope of aldehyde/ketone reaction partners are currently under investigation in addition to computer aided modeling and will be reported in due course. Note that a copy of the Annual Water Report for the Barwon Region, Victoria, Australia which includes the metal content of water stores in the region where this work was conducted can be accessed from the given web address in the Electronic Supplemenary Information.

## Supplementary Material



## Figures and Tables

**Table 1:** Ion solution used in aldol reaction from Scheme 2. [31]

Entry	Cation	Anion	Yield (%) a	dr (%) a*anti*/*syn*	ee (%) b
1 c	-	-	91	>99/1	>99
2 d	-	-	7	81/19	22
3	Na	F	49	83/17	67
4	Na	Cl	54	82/18	87
5	Na	Br	30	78/22	43
6	Na	I	17	79/21	34
7	Na	OAc	75	81/19	38
8	K	F	23	79/22	67
9	K	Cl	27	83/17	45
10	K	Br	44	80/20	35
11	K	I	32	75/25	21
12	Mg	Cl_2_	55	80/20	51
13	Ca	Cl_2_	99	45/55	2

a Determined by ^1^H NMR spectroscopy;

b Determined by chiral HPLC using a chiralpak AD-H Column;

c Deionised water used as a control;

d Tap water was used; Conditions: Water (1.6 mL) containing a saturated solution of the indicated salt was added to a round bottom flask charged with benzaldehyde (1 equivalent), ketone (5 equivalents) and catalyst 4 (1 mol%) stirred at room temperature for 24 h.

**Table 2:** Catalyzed aldol in transition metal salt solutions.

Entry	Cation	Anion	Yield (%) a	de (%) b*anti*/*syn*	ee (%) c
1	Cu	Cl	trace d	-	-
2	Cu	Br_2_	trace d	-	-
3	Fe(III)	Cl_3_	>99	42/58	0
4	Ni	Cl_2_	3	82/18	83
5	Zn	OAc	>99	80/20	92

a Isolated yield;

b Determined by ^1^H NMR spectroscopy;

c Determined by chiral HPLC using a chiralpak AD-H Column;

d Trace is defined here as <3% of desired product as determined by ^1^H NMR integration; Conditions: Water (1.6 mL) containing a saturated solution of the indicated salt was added to a round bottom flask charged with benzaldehyde (1 equivalent), ketone (5 equivalents) and catalyst 4 (1 mol%) stirred at room temperature for 24 h.

**Table 3:** Catalyzed aldol reaction of **5** and **6** in the presence of cation sequestering agent EDTA.

Entry	EDTA (% w/w)	Yield (%) a	de (%) a	ee (%) b
1	0	7	81	22
2 c	5	0	-	-
3	5	98	84	51

a Determined by ^1^H NMR spectroscopy;

b Determined by chiral HPLC using a chiralpak AD-H Column;

c No catalyst present; Conditions: Water (1.6 mL) containing a saturated solution of the indicated salt was added to a round bottom flask charged with benzaldehyde (1 equivalent), ketone (5 equivalents) and catalyst 4 (1 mol%) stirred at room temperature for 24 h.

**Table 4:** Alternative benzaldehydes and ketone used in the aldol reaction in aqueous solution.

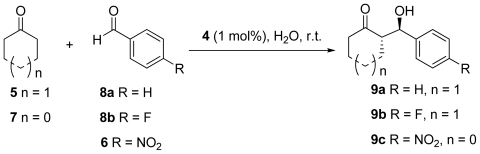						
Entry	Aldehyde	*n*	Salt	Conversion a	dr a (*anti*/*syn*)	ee b
1	**8a**	1	N/A c	45	92/8	68
2	**8a**	1	N/A d	82	84/16	28
3	**8a**	1	EDTA e	98	83/17	25
4	**8a**	1	Zn(OAc)_2_	44	76/24	4
5	**8b**	1	N/A c	21	91/9	92
6	**8b**	1	N/A d	80	86/14	68
7	**8b**	1	EDTA e	70	85/15	88
8	**8b**	1	Zn(OAc)_2_	70	76/24	64
9	**6**	0	N/A c	61	76/24	14
10	**6**	0	N/A d	56	39/61	22
11	**6**	0	EDTA e	74	34/66	13
12	**6**	0	Zn(OAc)_2_	97	29/71	14

a Determined by integration of key peaks in the ^1^H NMR spectrum;

b Determined by chiral HPLC, Chiral Pak AD-H column;

c Deionised water was used as the reaction medium;

d Tap water was used as the reaction medium;

e Tap water with EDTA (5%, w/w) was used as reaction medium; Conditions: Water (1.6 mL) containing a saturated solution of the indicated salt was added to a round bottom flask charged with benzaldehyde (1 equivalent), ketone (5 equivalents) and catalyst 4 (1 mol%) stirred at room temperature for 24 h.
