# Supplementary material for: Investigating Ionic Effects Applied to Water Based Organocatalysed Aldol Reactions
Source: Int J Mol Sci. 2011 Dec 7;12(12):9083–94. doi: 10.3390/ijms12129083 (PMC3257117; doi:10.3390/ijms12129083)
Supplement: Supplementary file 1 [file ijms-12-09083-s001.pdf]

Supplementary Information

1. General Experimental

All ^{1}H and ^{13}C NMR spectra were recorded on a Jeol JNM-EX 270 MHz or Jeol JNM-EX 400 MHz as indicated. Samples were dissolved in deuterated chloroform ($CDCl_3$) with the residual solvent peak used as an internal reference ($CDCl_3$–δH 7.26 ppm). Proton spectra are reported as follows: chemical shift δ (ppm), (integral, multiplicity (s = singlet, br s = broad singlet, d = doublet, dd = doublet of doublets, t = triplet, q = quartet, m = multiplet), coupling constant J (Hz), assignment).

Thin Layer Chromatography (TLC) was performed using aluminium-backed Merck TLC Silica gel 60 F254 plates, and samples were visualised using 254 nm ultraviolet (UV) light, and potassium permanganate/potassium carbonate oxidising dip (1:1:100 $KMnO_4$:K_2CO_3:H_2O w/w).

Column Chromatography was performed using silica gel 60 (70–230 mesh). All solvents used were AR grade. Specialist reagents were obtained from Sigma-Aldrich Chemical Company and used without further purification. Petroleum spirits refers to the fraction boiling between 40–60 °C.

Chiral HPLC was performed with a 1200 series Agilent. Separation of stereoisomers was carried out with a DiacelChiralpak AD-H chiral column (0.46 cm × 25 cm). Retention times were reported at ambient temp (24 °C) with an injection volume of 20 μL at a flow rate of 1 mL/min. A mobile phase of 10% isopropanol/90% hexane was used.

HRMS was found via a 6210 MSD TOF mass spectrometer under the conditions: gas temperature (350 °C), vaporizer (28 °C), capillary voltage (3.0 kV), cone voltage (40 V), nitrogen flow rate (7.0L/min), nebuliser (15 psi). Samples were dissolved in MeOH.

Specific rotation [α_D] was obtained using a JASCO DIPP Digital Polarimeter. Compounds were dissolved in $CHCl_3$ where indicated. Rotation was measured at λ = 584 nm and reported with the units 10^{-1} °C cm^2 g^{-1}.

Melting points were found on a Stuart Scientific Melting Point Apparatus SMP3, v.5 and are uncorrected.

The Annual Drinking water quality report for the region where this work was undertaken can be found at: http://www.barwonwater.vic.gov.au/image_get.cfm?id=A3102268

2. Synthesis of Bis-diprolinamide

2.1. trans-4-Hydroxy-N-Boc-L-proline 1

Trans-4-hydroxy-*L*-proline (3 g, 22.9 mmol) was solvated in 45 mL of THF/H_2O in a 2:1 ratio. NaOH (1.83 g, 45.8 mmol) was added to the solution and allowed to stir for 4 min. BocAnnhydride (6.5 g, 2.977 mmol) was added to the mixture and the solution was stirred for 24 h under a N_2 atmosphere. The resulting mixture was acidified with 2M HCl (50 mL) and the protected compound was extracted into Et_2O (3 × 60 mL). The solvent was removed *in vacuo* to give the protected species as a hygroscopic white foam (3.728 g, 70%). ^{1}H NMR (270 MHz, CDCl3): δ (ppm): 4.43 (br. m, 2H, NH-C*H*-CH_2-C*H*), 3.51 (br. m, 2H, NH-CH_2), 2.26 (br. m, 1H, CH-CH_2-CH), 2.09 (br. m, 1H, CH-CH_2-CH), 1.41 (d, 9H, *J* = 13 Hz, *t*-butyl). The compound was identified by ^{1}H NMR and used without further purification.

2.2. trans-4-Hydroxy-N-Boc-L-proline Benzyl Ester

Benzyl bromide (1.43 mL, 11.8 mmol) was added to a solution of 4-OH-Boc-Proline **1** (2.483 g, 10.7 mmol) in THF and cooled to 0 °C. Triethylamine (1.65 mL, 11.8 mmol) was added and the resulting mixture was stirred for 18 h, gradually reaching room temperature. The solvent was subsequently removed *in vacuo* and the crude mixture was redissolved in DCM. The solution was washed with 1M HCl (2 × 30 mL), brine (2 × 30 mL), Na_2CO_3 (2 × 30 mL) and an additional wash of brine (1 × 30 mL). The washed organic phase was dried over $MgSO_4$ which was filtered. The solvent was removed *in vacuo* to give the crude product as a viscous, pale yellow oil. The crude product was purified by column chromatography using gradient elution (2:1 pet. spirits: EtOAc→ 100% EtOAc) to give the benzyl protected species as a pale yellow oil (2.17 g, 67%), R_f : 0.21 (2 pet. spitis:1 EtOAc); ^{1}H NMR (270 MHz, $CDCl_3$): δ (ppm) 7.72 (br s, 5H, aryl), 5.04–5.17 (m, 2H, C*H*$_2$Ph), 4.42–4.30 (m, 2H, αH), 3.68–3.49(m, 2H, CH-C*H*$_2$-CH), 2.20–1.97 (m, 2H, N-C*H*$_2$-CH), 1.39–1.27 (m, 9H, -*t*-butyl); $[\alpha]_D^{20.7}$ = −61.1° (c = 0.118, $CHCl_3$). The spectra was consistent with data previously reported.[1].

2.3. trans-4-tert-Butyldiphenylsiloxy-N-Boc-L-proline Benzyl Ester (Analysis of Analytically Pure Sample Obtained for Characterisation)

Benzyl protected proline (0.6726 g, 2.1 mmol) was dissolved in DMF (15 mL). Imidazole (0.57 g, 8.38 mmol), DMAP (0.102 g, 0.838 mmol) and *tert*-butyldiphenylsilyl chloride (0.928 mL, 2.31 mmol) were added to the solution and the mixture was stirred for 24 h. The reaction was quenched with cold H_2O (100 mL) and acidified with 2 M HCl (50 mL). The final product was extracted into EtOAc (3 × 30 mL), the combined organic phase was washed with 2 M HCl (3 × 30 mL) and saturated $NaHCO_3$(3 × 30 mL). The washed organic phase was dried over $MgSO_4$ and the solvent was removed *in vacuo* to give the silylated species as a thick resin which was used without further purification. An analytically pure sample was obtained for characteriation purposes via flash chromatography (1:5 EtOAc, Pet Spirits) to give **3** as a colourless oil (0.673 g, 57%). R_f = 0.52; ^{1}H NMR (Figure S1, 270 MHz, $CDCl_3$) δ (ppm): 7.61 (m, 4H, aryl), 7.37 (m, 10H, aryl), 5.11 (m, 2H, Bn), 4.423 (m, 2H, N-C*H*-CH_2-C*H)*, 3.48 (m, 2H, N-C*H*$_2$), 2.26 (m, 1H, CH-C*H*$_2$-CH), 1.88 (m, 1H, CH-C*H*$_2$-CH), 1.42 (d, *J* = 23 Hz, 9H, Si-C(CH_3)$_3$), 1.05 (s, 9H, Si-C(CH_3)$_3$); ^{13}C NMR (Figure S2, 100 MHz, $CDCl_3$) δ (ppm) = 172.86, 154.59, 135.57, 133.89, 129.87, 128.55, 128.43, 127.76, 80.03, 71.41, 70.65, 66.64, 58.27, 57.83, 54.70, 54.37, 39.51, 38.67, 28.37, 28.18, 26.77, 19.02; $[\alpha]_D^{20.7}$ = −37.2° (c = 0.123, $CHCl_3$); λ_{max} = 1747 (s), 1427 (s), 1175 (s), 1105 (s); HRMS calculated for $[C_{33}H_{41}NO_5SiNa]^+$ M = 582.26462 found *m*/*z* = 582.26402.

Figure S1. ^{1}H NMR spectrum for *Trans-4-tert-butyldiphenylsiloxy-N-Boc-*L*-proline benzyl ester.*

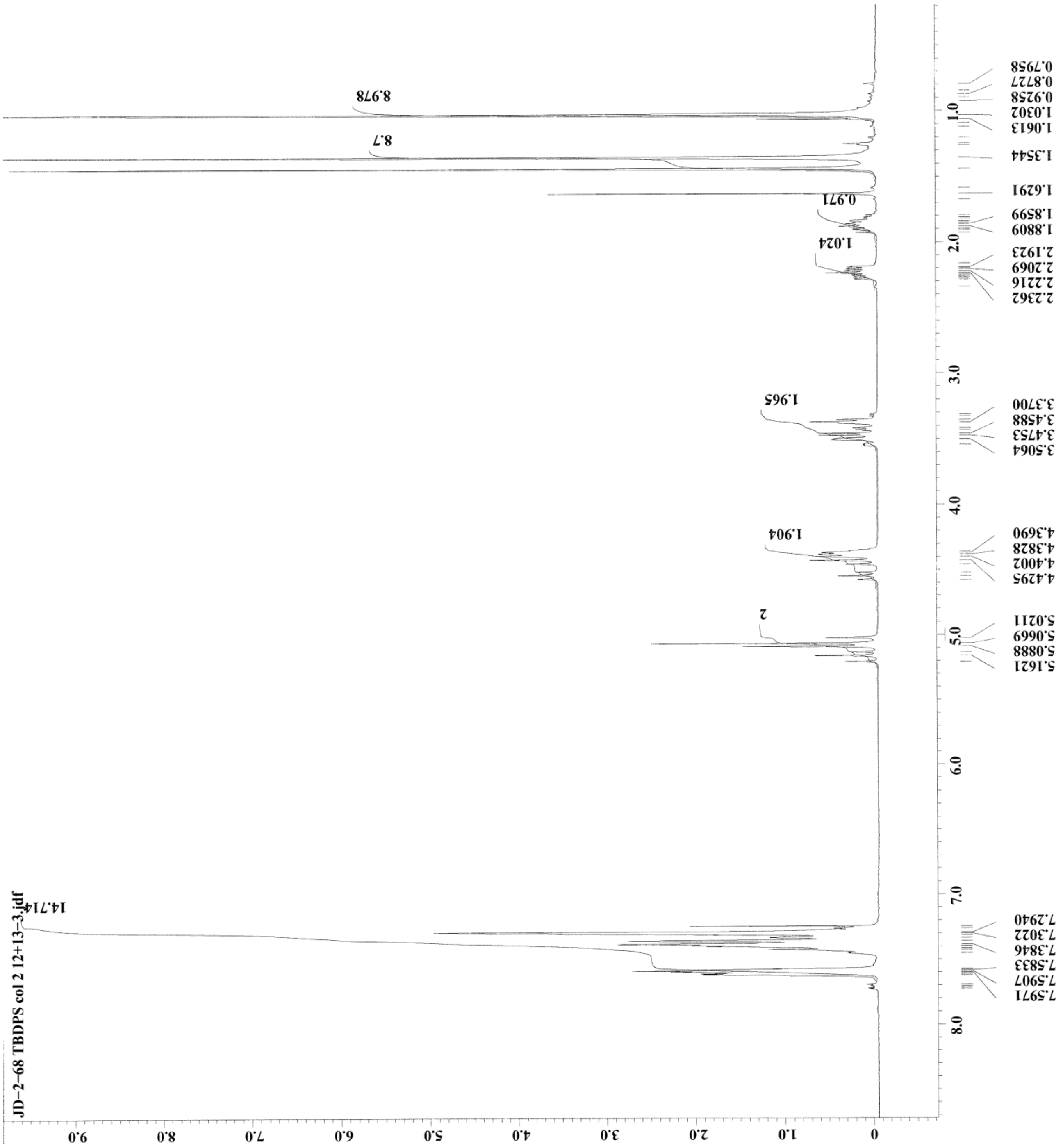

Figure S2. ^{13}C NMR for *Trans-4-tert-butyldiphenylsiloxy-N-Boc-*L*-proline benzyl ester.*

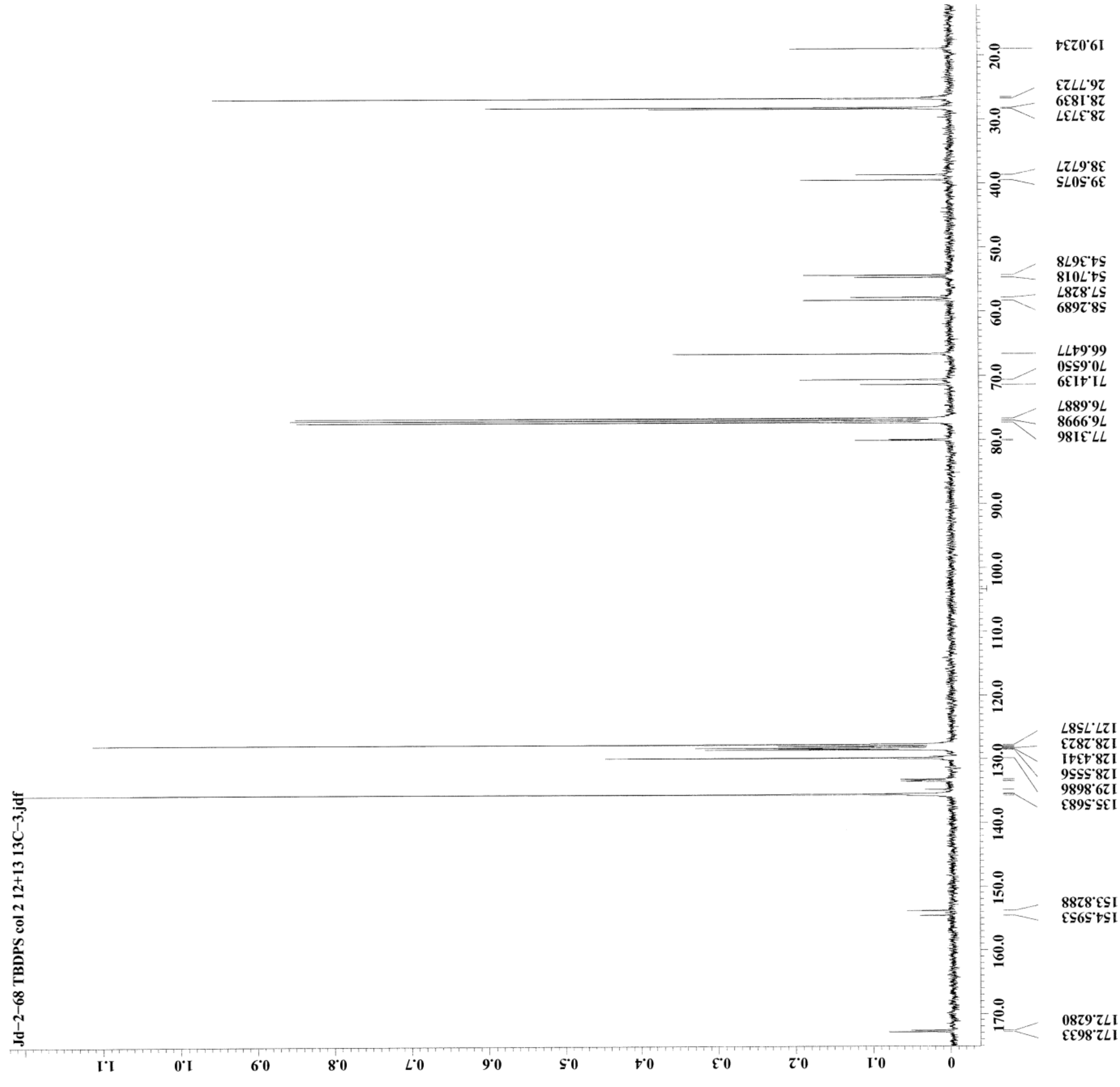

*2.4. trans-4-tertButyldiphenylsiloxy-N-Boc-*L*-proline Carboxylate* **2**

Silylatedproline(8.9 g) was dissolved in MeOH (20 mL) and Pd/C (0.89 g, 10% w/w) was added. The mixture was stirred under H_2 (balloon) for 18 h and the resulting solution was vacuum filtered through celite and the filtrate evaporated under reduced pressure. The crude product was purified by column chromatography (1:9 EtOAc/pet. Spirits) to give the deprotected acid **2** as a viscous, pale brown oil (1.217 g, 72% over two steps). R_f : 0.22 (1/3; EtOAc/Pet. Spirits); ^{1}H NMR (Figure S3, 270 MHz, $CDCl_3$) δ (ppm): 7.6 (m, 4H, aryl), 7.39 (m, 6H aryl), 4.52 (t, 1H, J = 5.18 Hz, C*H*-CO), 4.4 (m, 1H, CH_2-C*H*-CH_2), 3.52 (m, 1H, N-CH_2), 3.44 (m, 1H, N-CH_2), 2.25 (m, 1H, CH-CH_2-CH), 2.06 (br m, 1H, CH-CH_2-CH), 1.42 (d, 9H, J = 12.6 Hz, Si-C(CH_3)$_3$), 1.05 (s, 9H, Si-C(CH_3)$_3$); ^{13}C NMR (Figure S4, 100 MHz, $CDCl_3$) δ (ppm) = 157.5, 135.6, 133.8, 130, 127.8, 82.1, 70.8, 55.0, 54.5, 39.5,

37.3, 28.3, 26.8, 19.0; [α] $_D^{20.3}$ = –41.9° (0.126, $CHCl_3$). λ_{max}= 1748 (s), 1472 (s), 1105 (s); HRMS calculated for $[C_{26}H_{35}NO_5SiNa]^+$ M = 492.21767 found *m*/*z* = 492.21804.

Figure S3. ^{1}H NMR for *trans-4-tertbutyldiphenylsiloxy-N-Boc-*L*-proline carboxylate* **2.**

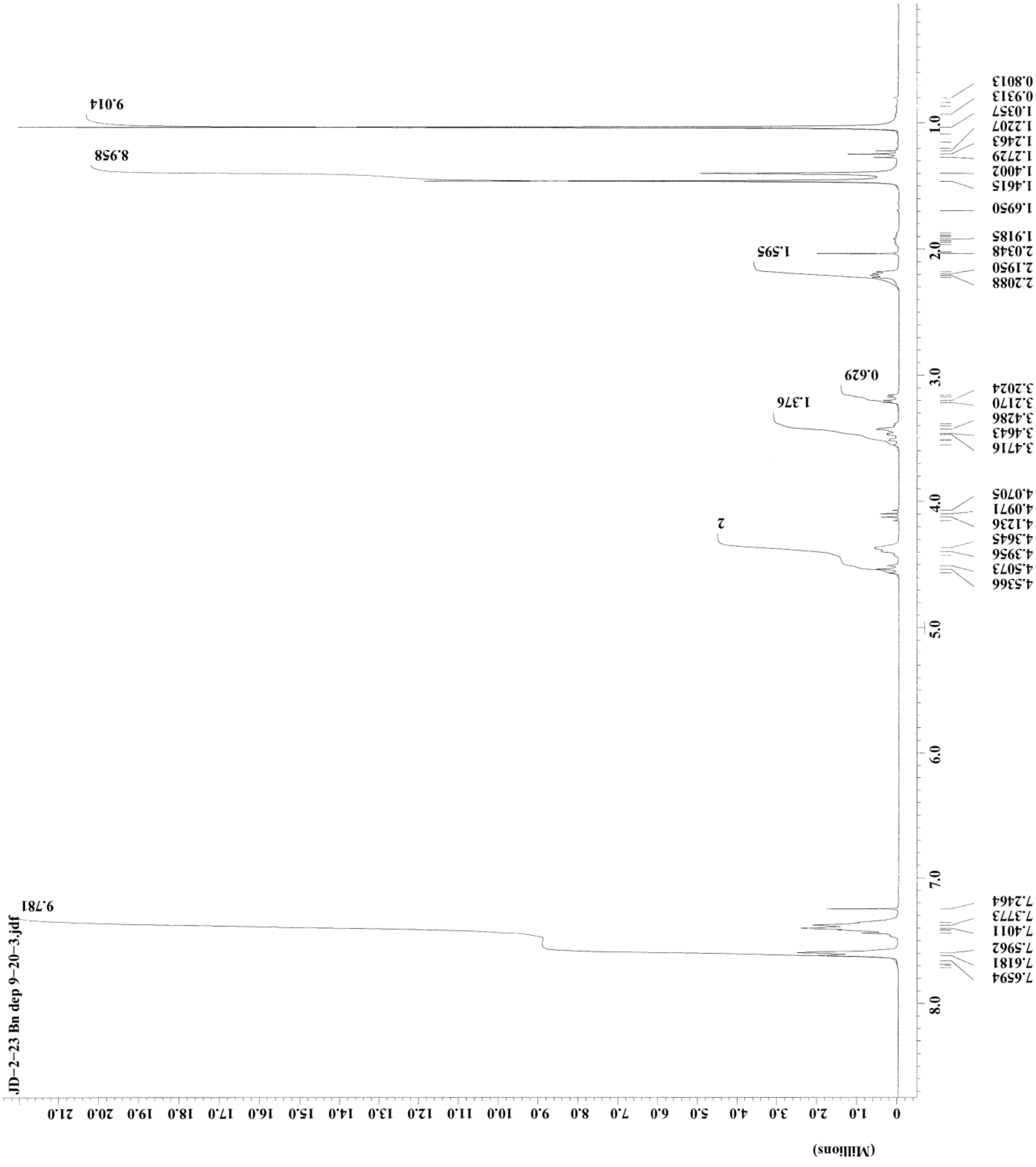

Figure S4. ^{13}C NMR for *trans-4-tertbutyldiphenylsiloxy-N-Boc-*L*-proline carboxylate* **2**.

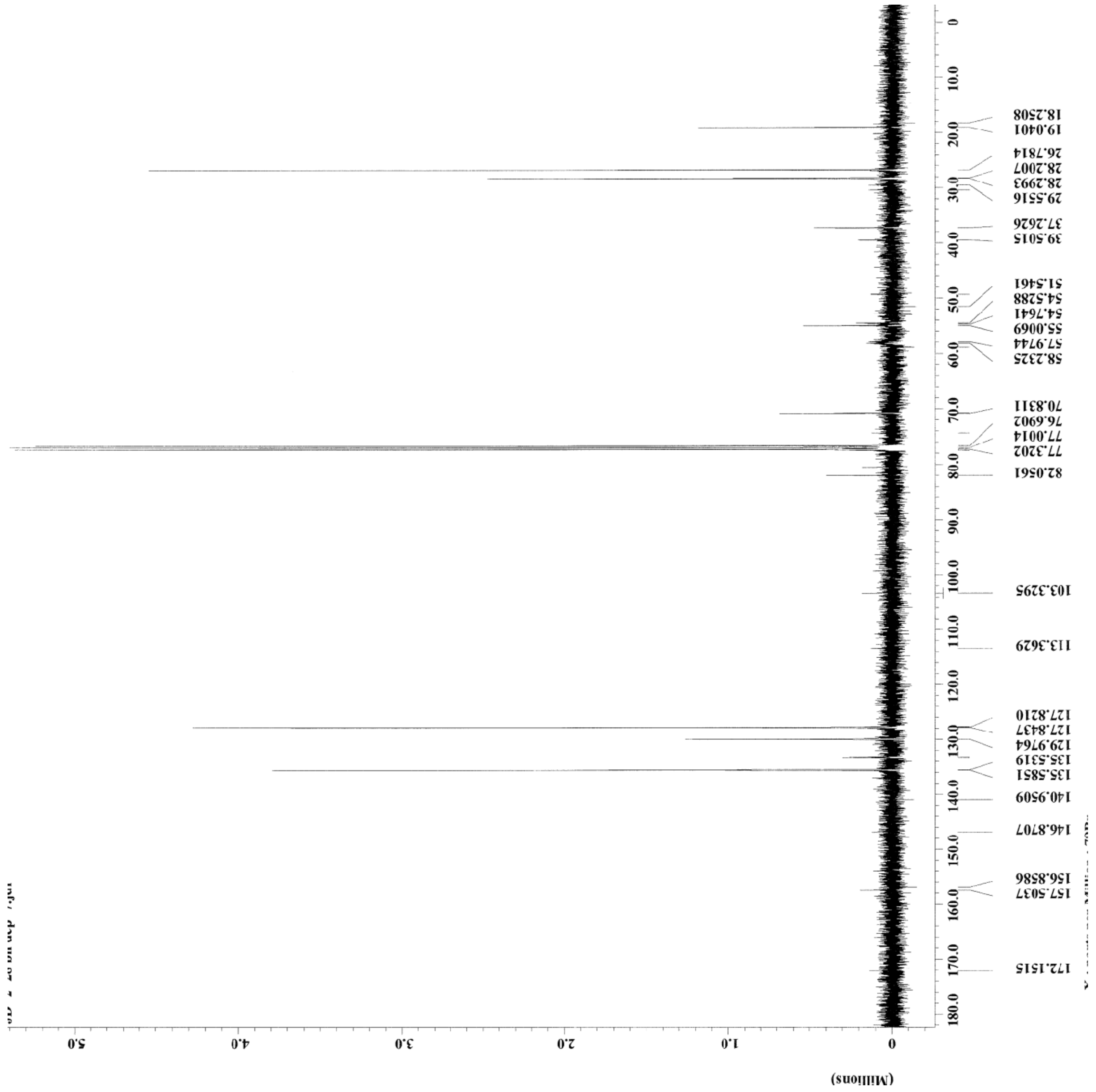

*2.5. 1,6-di(trans-N-Boc-4-tertbutlydiphenylsiloxy-*L*-prolinamide) Hexane* **3**

TBDPSO proline **2** (0.714 g, 2.34 mmol) was dissolved in 15 mL DCM and cooled to 0 °C. HOBt (0.072 g, 0.533 mmol) was added to the mixture and stirred for 4 min before the addition of EDCI (0.269, 1.41 mmol).To this mixture 1,6-diaminohexane (0.139 g, 1.16 mmol) was added and theresulting solution was allowed stirred for 16 h,allowing to reach room temperature. The final mixture was diluted with addition DCM (50 mL) and washed with 2M HCl (2 × 30 mL), Na_2CO_3 (2 × 30 mL) and brine (2 × 30 mL). The solvent was removed *in vacuo* and the crude product was purified by column chromatography (2:1 Pet. Spirits: EtOAc) to afford the *N*-boc diprolinamide as a white amorphous solid (0.484 g, 80%). m.p. = 67–68 °C. R_f : 0.14; ^{1}H NMR (Figure S5, 270 MHz, $CDCl_3$) δ (ppm) = 7.59–7.25 (m, 20H aryl), 4.39 (m, 4H, chiral H), 3.69–3.42 (m, 4H, CH-CH_2-CH), 3.17 (br s, 4 H, CH_2-HN), 2.39–1.97 (br m, 4H, N-CH_2), 1.43 (s, 18H, *Boc*), 1.40 (br, 4H, alkyl), 1.24

(br, 4H, alkyl), 1.02 (s, 18H, Si-C(CH_3)$_3$); ^{13}C NMR (Figure S6, 100 MHz, $CDCl_3$) δ (ppm) = 172.58, 156.26, 135.72, 135.66, 129.94, 127.86, 80.52, 71.62, 71.12, 58.9, 55.18, 40.24, 39.21, 27.23, 28.42, 26.89, 26.31, 19.15. $[\alpha]_D^{20.7} = -49°$ (c = 0.051, $CHCl_3$); λ_{max}= 1662 (s), 1472 (s); HRMS calculated for $[C_{52}H_{82}N_4O_8Si_2Na]^+$ M = 1041.55634 found *m*/*z* = 1041.55572.

Figure S5. ^{1}H NMR for *1,6-di(trans-N-Boc-4-tertbutlydiphenylsiloxy-*L*-prolinamide) hexane* **3**.

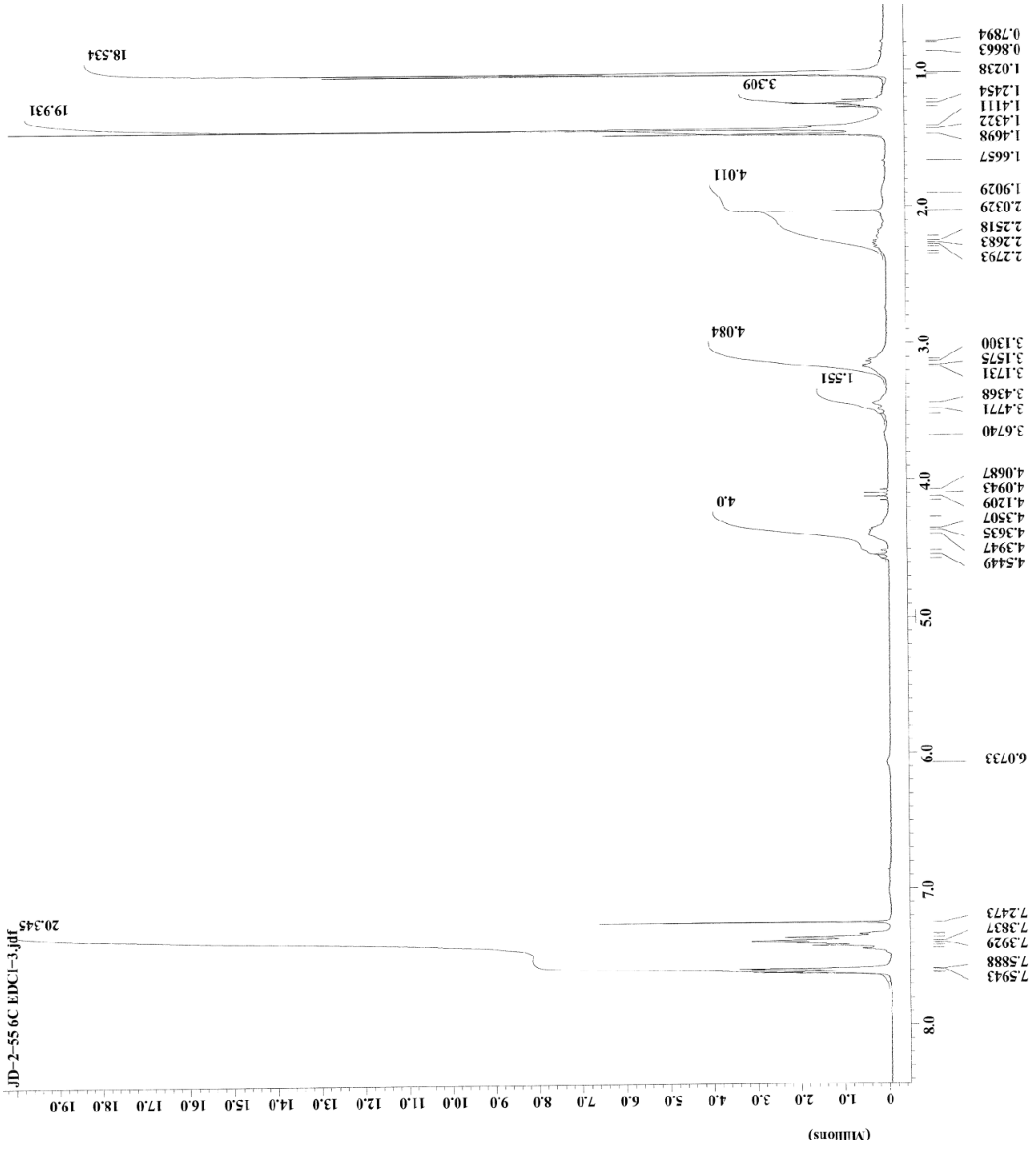

Figure S6. ^{13}C for *1,6-di(trans-N-Boc-4-tertbutlydiphenylsiloxy-L-prolinamide) hexane* **3**.

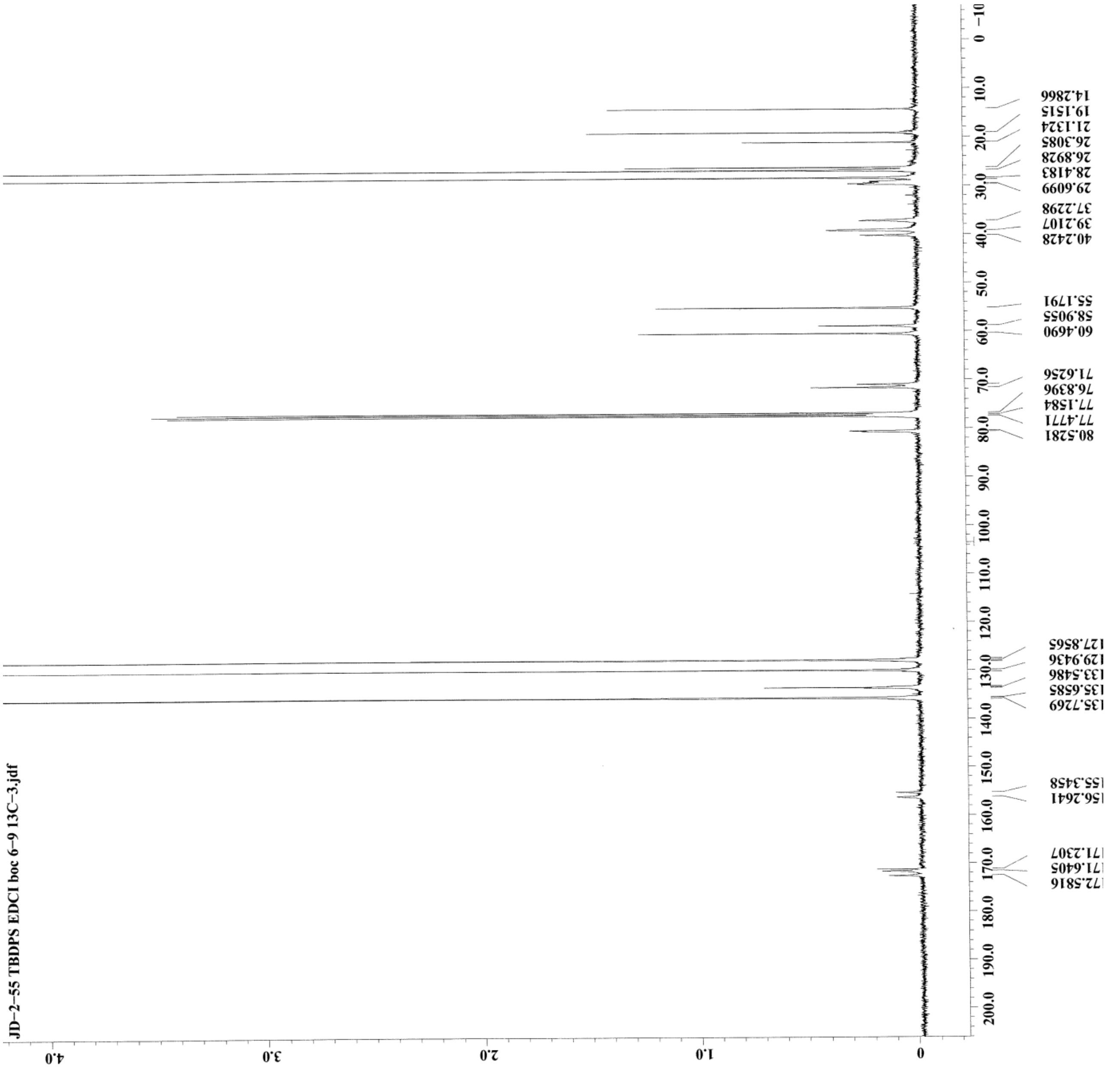

2.6. 1,6-di(trans-4-tertButlydiphenylsiloxy-L-prolinamide) Hexane

Boc protected dimer **3** (0.318 g, 0.312 mmol) was stirred in a solution of 10% trifluoroacetic acid (0.5 mL) in DCM (4.5 mL) for 4 hours under a N_2 atmosphere. The resulting solution was dissolved in additional DCM (20 mL) and basified with saturated $NaHCO_3$ (30 mL). The deprotected compound was extracted into DCM (3 × 30 mL) and the combined organic phase was washed with $NaHCO_3$ (3 × 30 mL). The solvent was removed *in vacuo* to give **4** as a pale brown solid. (0.252 g, 99%). R_f : 0.25 (100%, EtOAc); M.p. = 60–61 °C; ^1H NMR (Figure S7, 270 MHz, $CDCl_3$) δ (ppm) = 7.608 (br m, 8H, aryl), 7.38 (br m, 12H, aryl), 4.35 (br, 2H, CH_1-O), 3.99 (t, *J* = 8.4 Hz, CH_1-N), 3.14 (sept, 4H, CH_2-N),2.89 (d, *J* = 12.1 Hz, 2H, N-CH_2-CH), 2.56 (dd, *J* = 4.45, 7.7 Hz, 2H, N-CH_2-CH), 2.24 (m, 2H, CH-CH_2-CH), 1.7 (m, 2H, CH-CH_2-CH), 1,41 (m, 4H, alkyl), 1.27 (m, 4H, alkyl), 1.03 (br s, 18H, $C(CH_3)_3$); ^{13}C NMR (Figure S8, 100 MHz, $CDCl_3$) δ (ppm) = 174.6, 135.7, 134.1, 129.9, 127.8, 75.1,

56.9, 55.7, 40.1, 38.7, 29.6, 27, 26.53, 19.2; $[\alpha]_D^{20.7} = -137.5°$ (c = 0.024, $CHCl_3$); λ_{max}= 1654 (s), 1205 (s), 699 (s); HRMS calculated for $[C_{48}H_{67}N_4O_4Si_2H]^+$ M = 819.46954 found *m*/*z* = 819.46880.

Figure S7. ^{1}H NMR for *1,6-di(trans-4-tertbutlydiphenylsiloxy-L-prolinamide) hexane.*

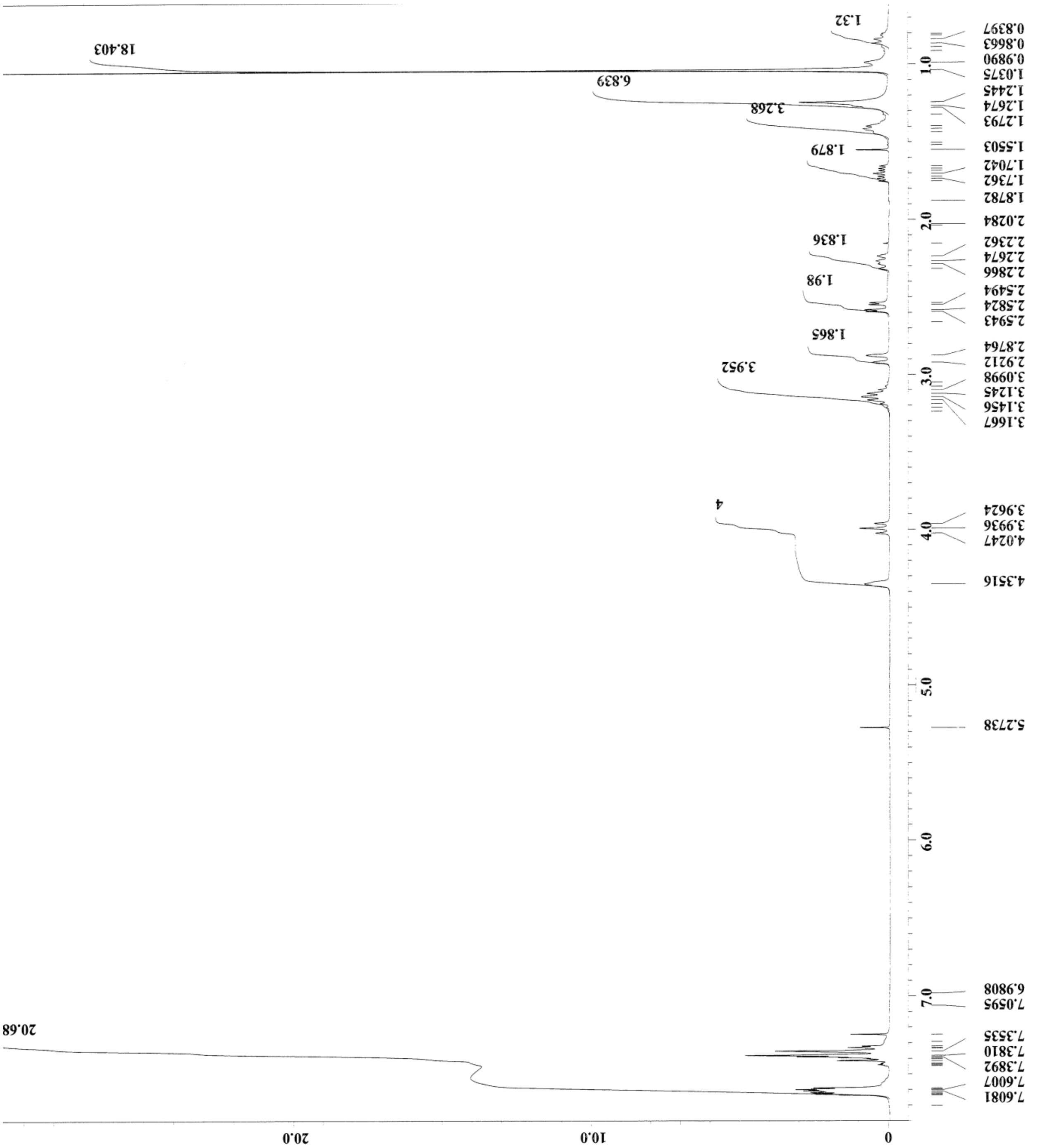

Figure S8. ^{13}C NMR for *1,6-di(trans-4-tertbutlydiphenylsiloxy-L-prolinamide) hexane.*

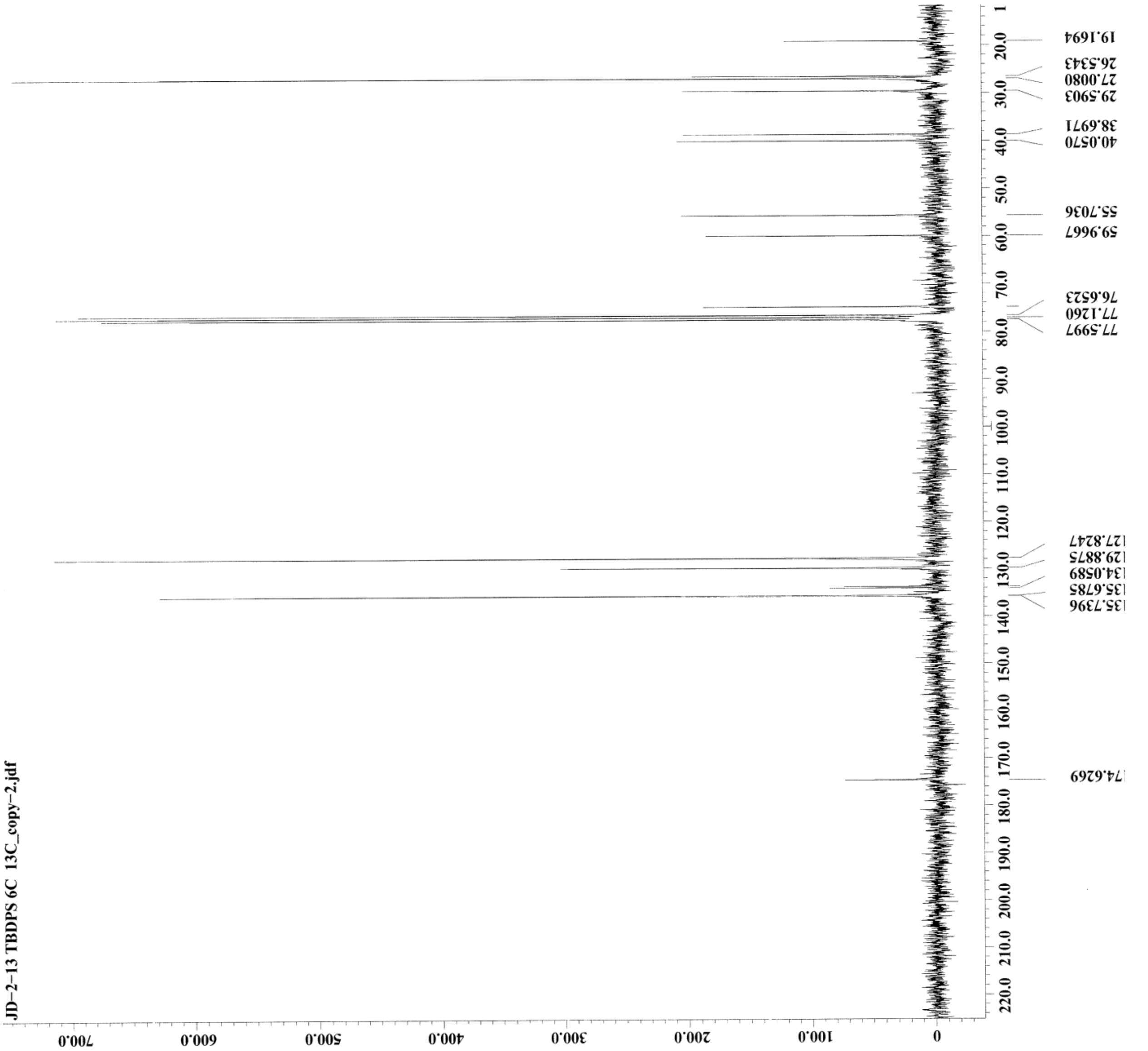

3. Determination of Conversion and Diastereomeric Ratio via ^{1}H NMR

The conversion of the initial aldehyde into the target compound was determined by the integration of key peaks within the ^{1}H NMR spectra (Figure S9). The diasteremeric ratio was determined by integration of the chiral proton peaks for both the *syn* and the *anti*diastereomer.

Figure S9. Worked example of determining conversion and dr.

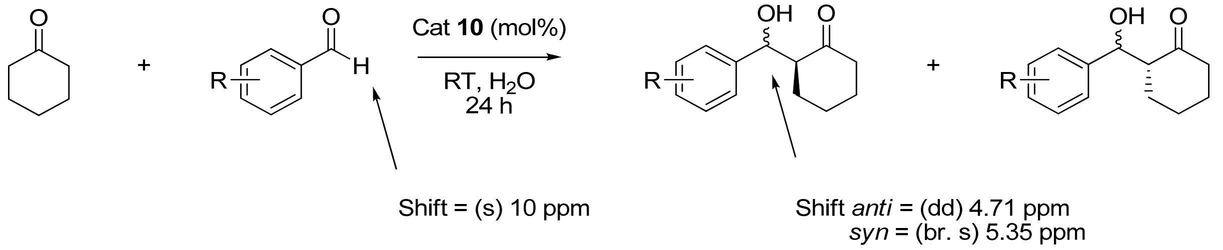

Figure S9. *Cont.*

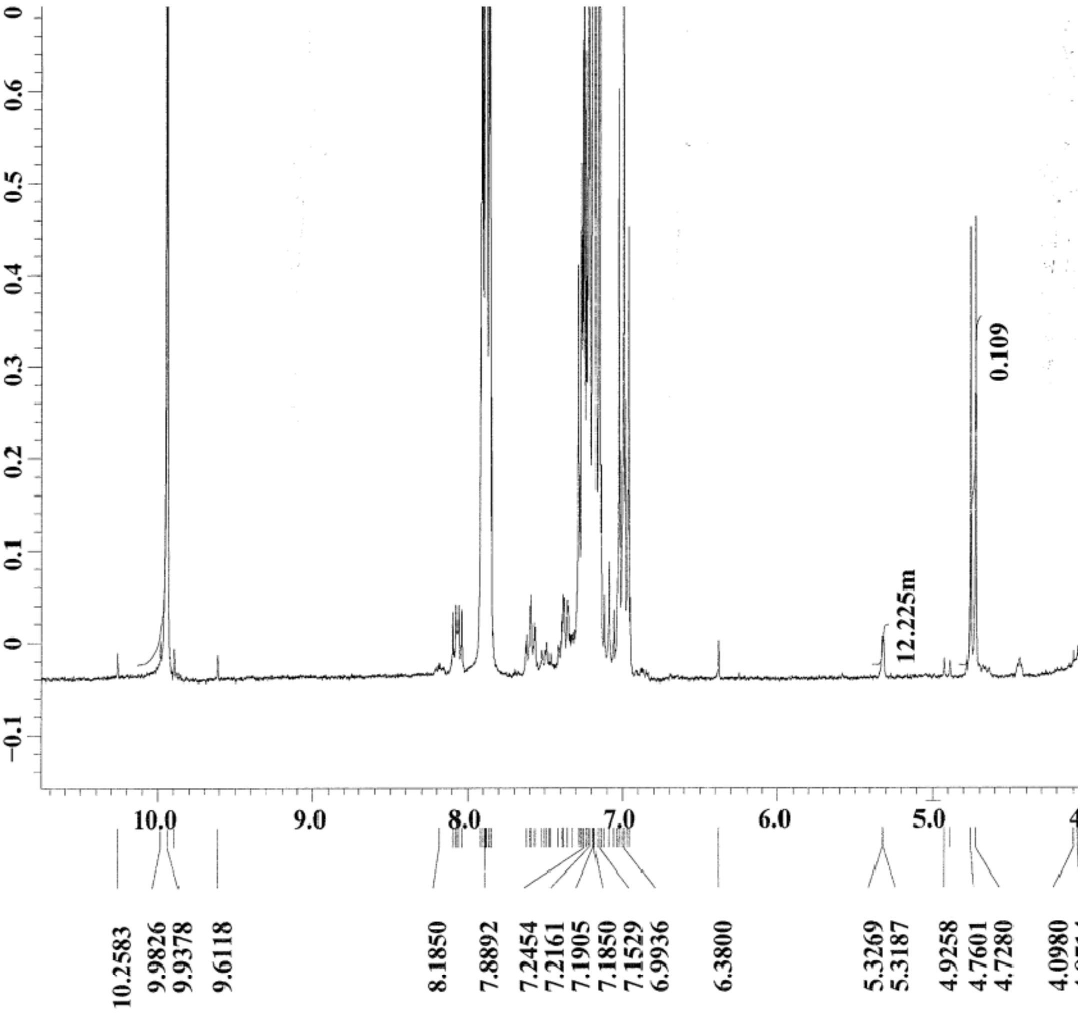

4. Determination of Enantiomeric Excess via Chiral HPLC

The enantiomeric excess was determined through the integration of each enantiomer investigating ionic effects applied to water based organocatalysedaldol reactions.

The chiral HPLC traces for each of the reactions carried out within the manuscript and their corresponding racemate standards are given here:

Table 1, entries 3–13 please refer to Figures S10–S23.

Table 2, entries 3–5 please refer to Figures S24–S26.

Table 3, entry 3 please refer to Figure S27.

Table 4, entries 1–12 please refer to Figures S28–S42.

Figure S10. 2-[Hydroxy-(4-Nitro-phenyl)-methyl]-cyclohexanone.

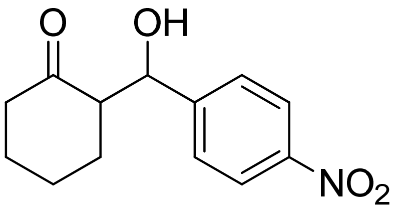

Racemic

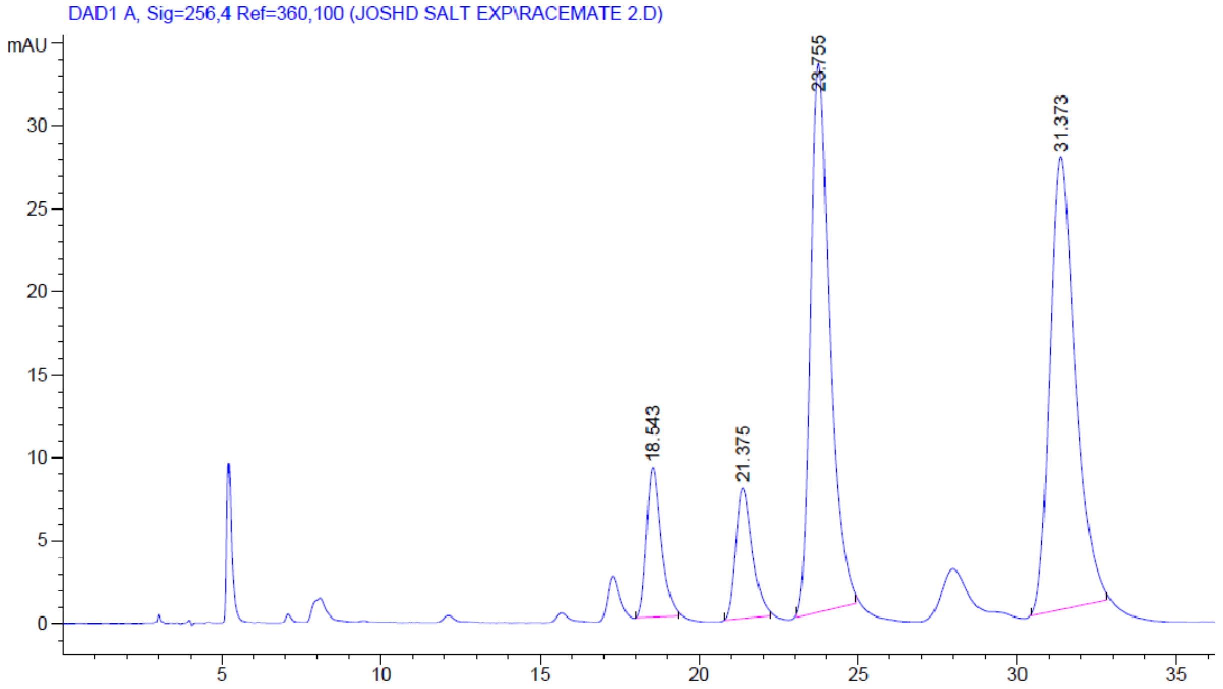

Signal 1: DAD1 A, Sig=256,4 Ref=360,100

Peak #	RetTime [min]	Type	Width [min]	Area [mAU*s]	Height [mAU]	Area %
1	18.543	BB	0.4741	286.97958	8.99672	8.2210
2	21.375	BB	0.5344	286.54929	7.87652	8.2087
3	23.755	BB	0.6277	1394.35535	33.08278	39.9437
4	31.373	BB	0.8336	1522.91663	27.26819	43.6266
Totals :				3490.80084	77.22421	

Figure S11. Deionized water (Table 1. Entry 1).

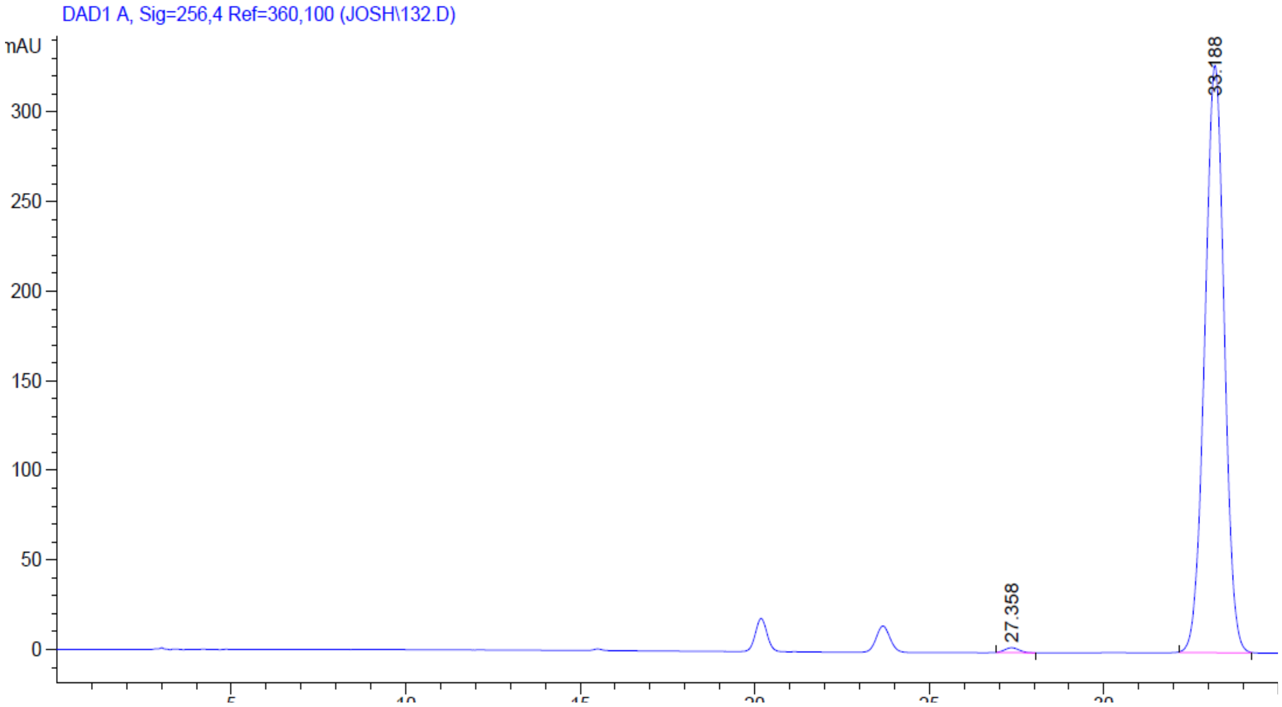

Signal 1: DAD1 A, Sig=256,4 Ref=360,100

Peak #	RetTime [min]	Type	Width [min]	Area [mAU*s]	Height [mAU]	Area %
1	27.358	BB	0.4265	77.65517	2.72491	0.6048
2	33.188	BB	0.6060	1.27614e4	327.92426	99.3952

Totals :				1.28390e4	330.64916	

Figure S12. Tap water (Table 1. Entry 2).

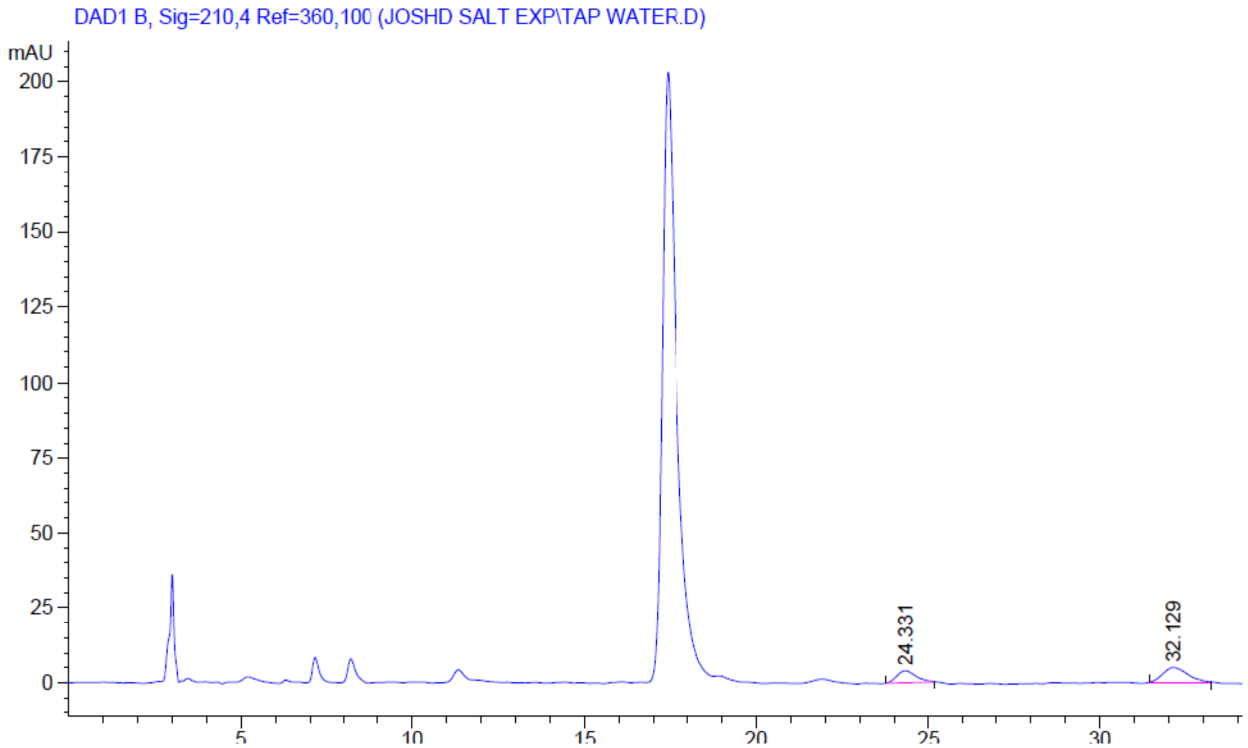

Signal 1: DAD1 B, Sig=210,4 Ref=360,100

Peak #	RetTime [min]	Type	Width [min]	Area [mAU*s]	Height [mAU]	Area %
1	24.331	BB	0.5401	155.29146	4.00709	39.2519
2	32.129	BB	0.6301	240.33600	4.86722	60.7481
Totals :				395.62746	8.87431	

Figure S13. NaF (Table 1. Entry 3).

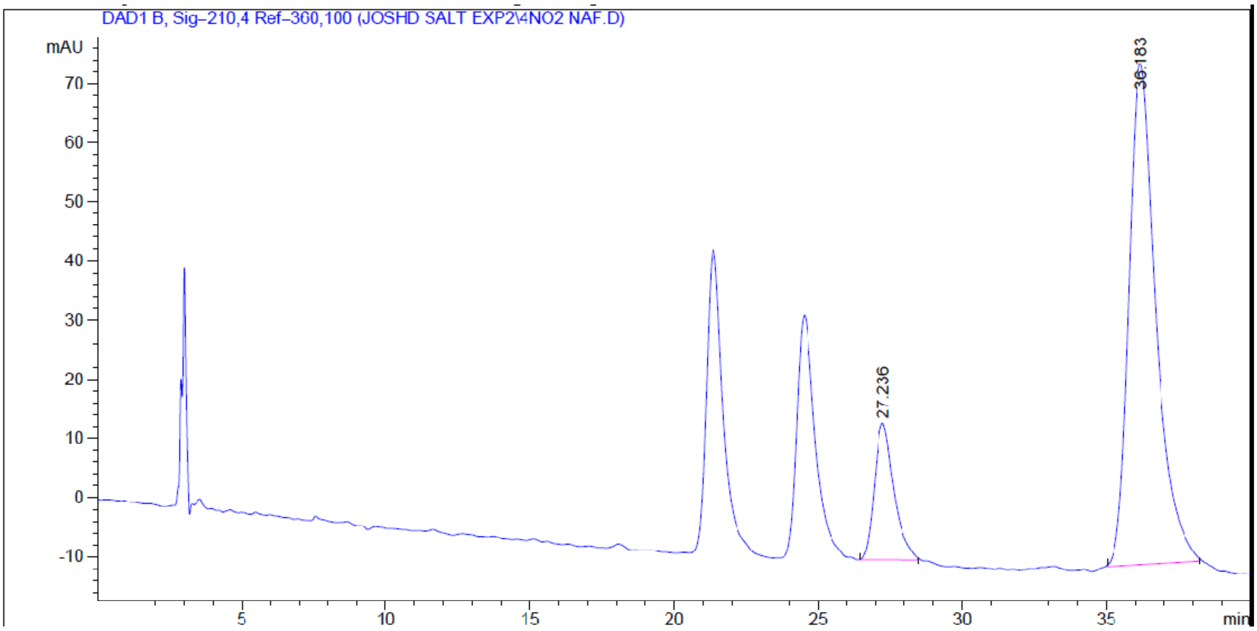

Signal 1: DAD1 B, Sig=210,4 Ref=360,100

Peak #	RetTime [min]	Type	Width [min]	Area [mAU*s]	Height [mAU]	Area %
1	27.236	BB	0.6895	1077.04529	23.12055	16.2939
2	36.183	BB	0.9799	5533.05908	84.72364	83.7061

Totals : 6610.10437 107.84419

Figure S14. NaCl (Table 1. Entry 4).

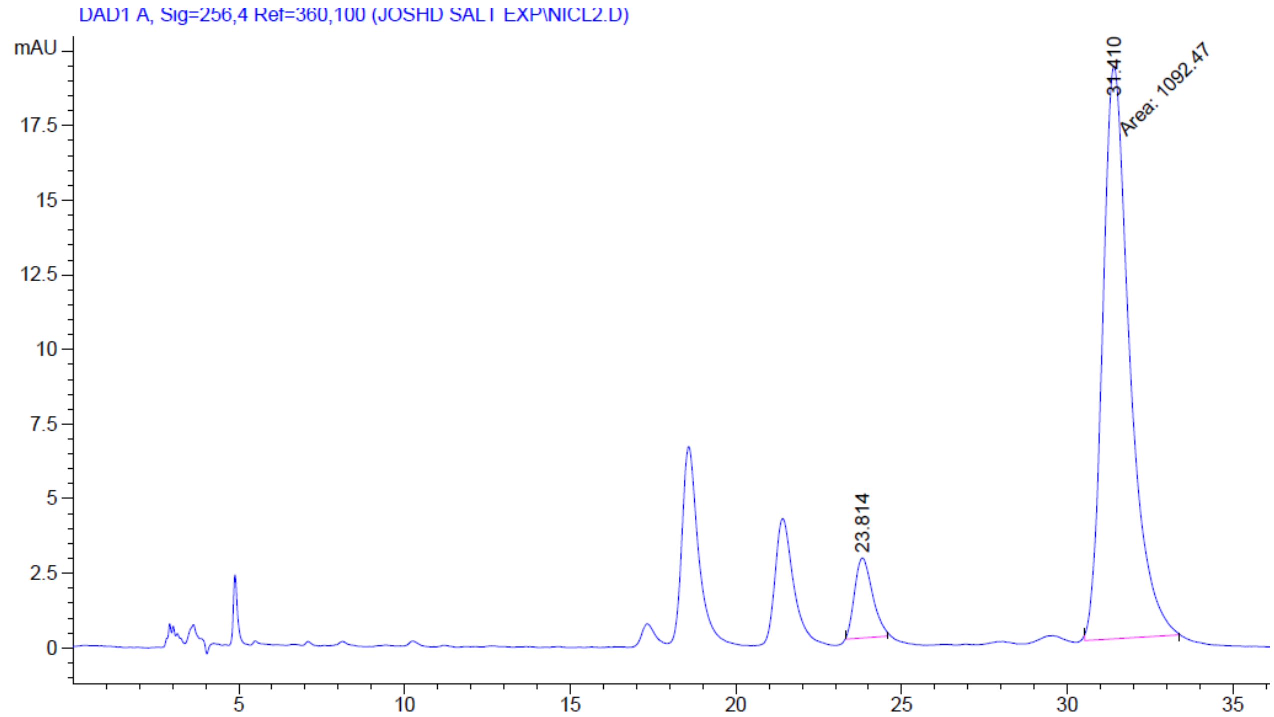

Signal 1: DAD1 A, Sig=256,4 Ref=360,100

Peak #	RetTime [min]	Type	Width [min]	Area [mAU*s]	Height [mAU]	Area %
1	23.814	BB	0.5484	100.19231	2.67867	8.4007
2	31.410	MM	0.9487	1092.46948	19.19181	91.5993

Totals : 1192.66179 21.87049

Figure S15. NaBr (Table 1. Entry 5).

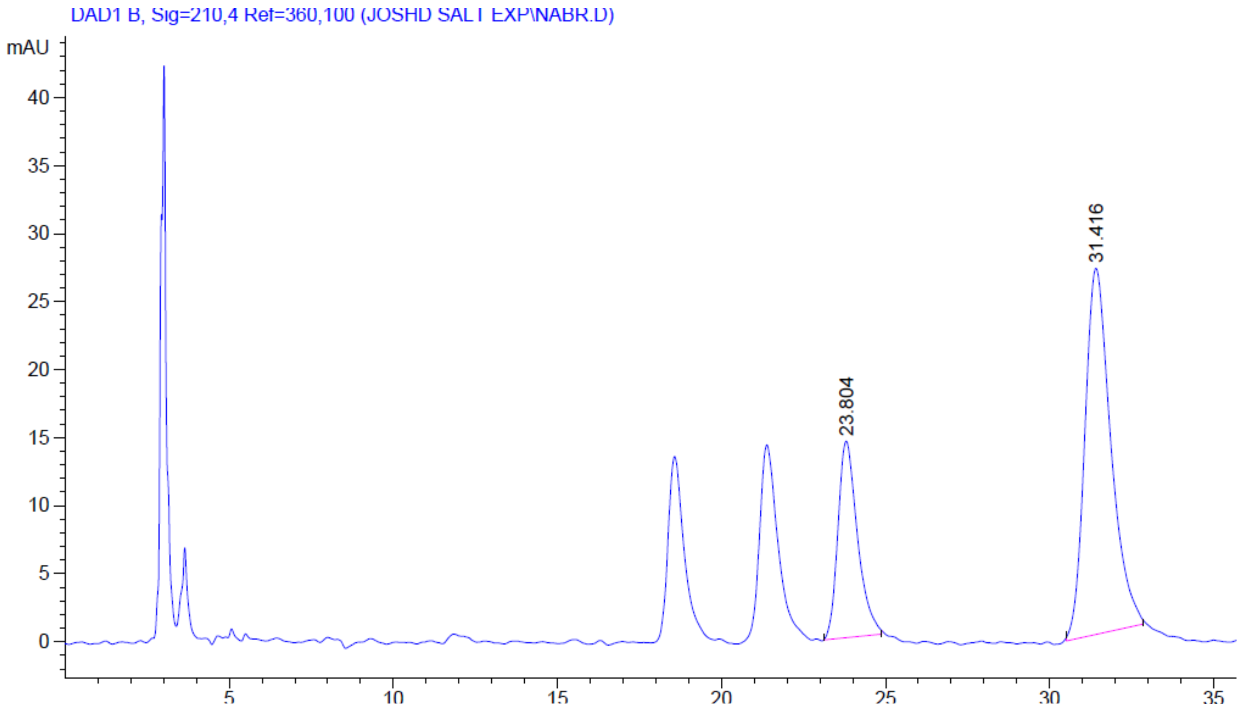

Signal 1: DAD1 B, Sig=210,4 Ref=360,100

Peak #	RetTime [min]	Type	Width [min]	Area [mAU*s]	Height [mAU]	Area %
1	23.804	BB	0.6119	599.31458	14.44799	28.7730
2	31.416	BB	0.8010	1483.59204	26.91704	71.2270

Totals : 2082.90662 41.36503

Figure S16. NaI (Table 1. Entry 6).

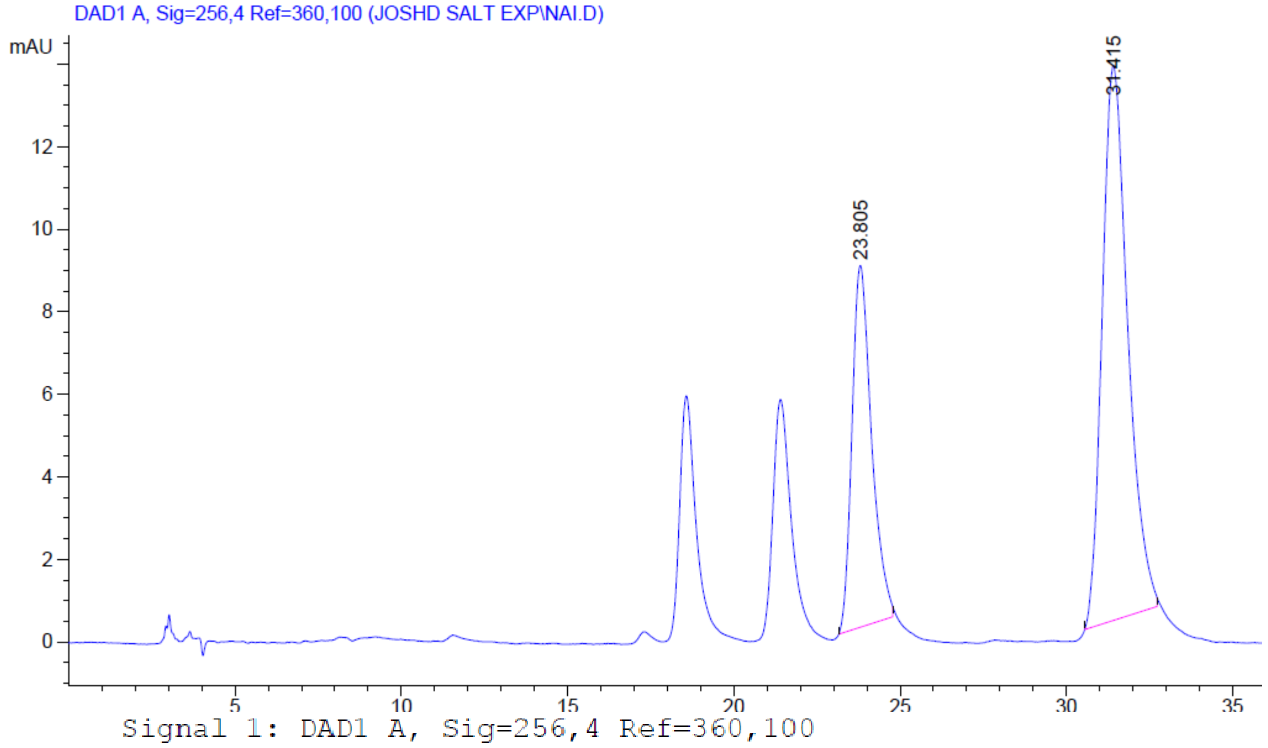

Signal 1: DAD1 A, Sig=256,4 Ref=360,100

Peak #	RetTime [min]	Type	Width [min]	Area [mAU*s]	Height [mAU]	Area %
1	23.805	BB	0.6099	359.02625	8.76449	33.2814
2	31.415	BB	0.7865	719.73309	13.44572	66.7186
Totals :				1078.75934	22.21022	

Figure S17. NaOAc (Table 1. Entry 7).

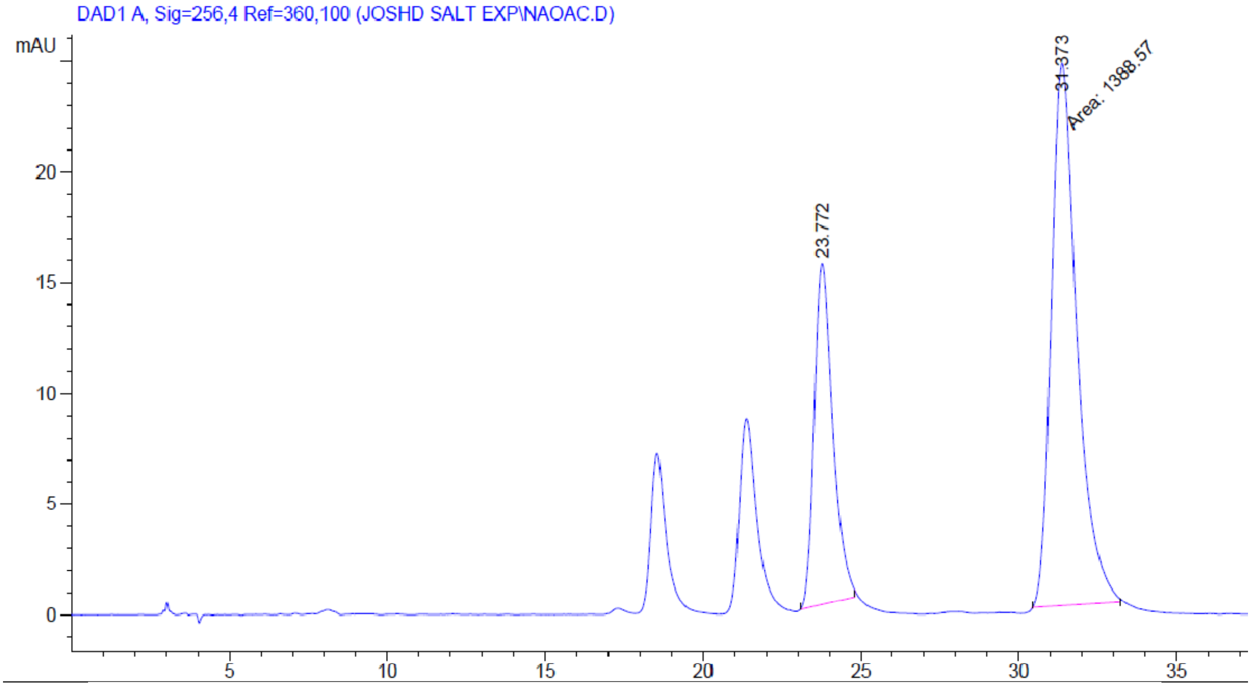

Signal 1: DAD1 A, Sig=256,4 Ref=360,100

Peak #	RetTime [min]	Type	Width [min]	Area [mAU*s]	Height [mAU]	Area %
1	23.772	BB	0.6176	629.53204	15.37830	31.1943
2	31.373	MM	0.9454	1388.56677	24.47832	68.8057
Totals :				2018.09882	39.85662	

Figure S18. KF (Table 1. Entry 8).

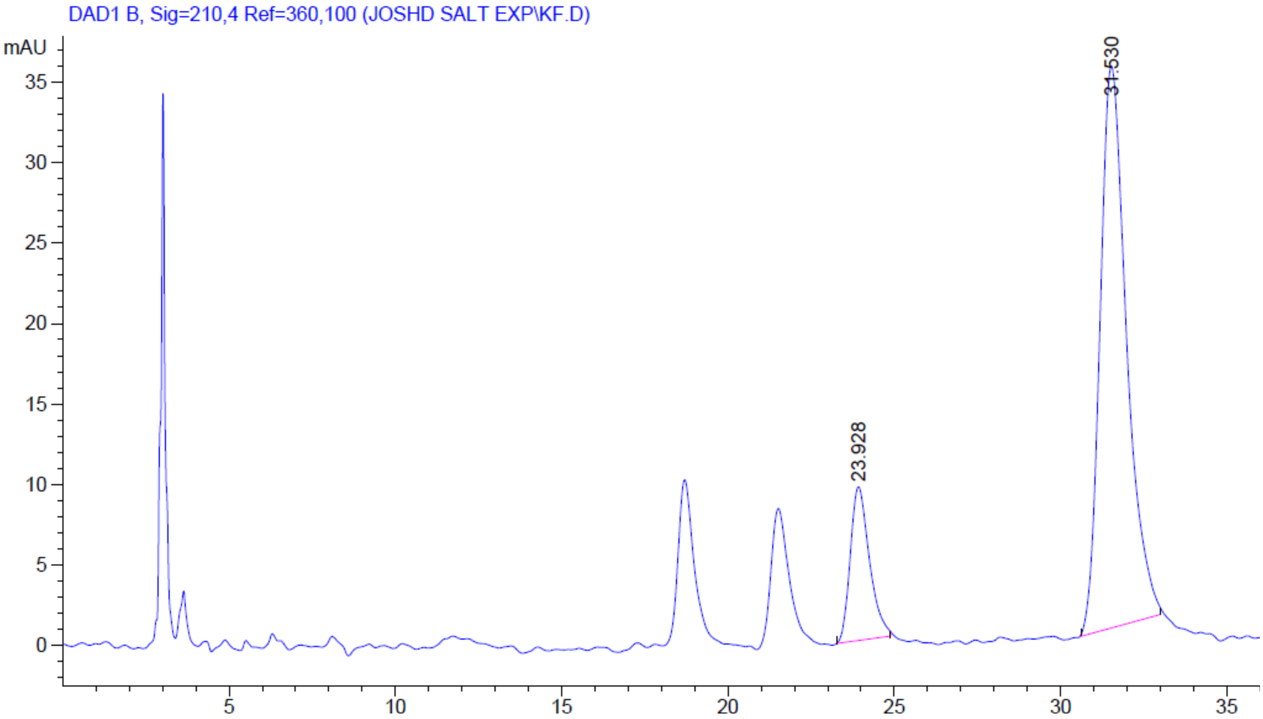

Signal 1: DAD1 B, Sig=210,4 Ref=360,100

Peak #	RetTime [min]	Type	Width [min]	Area [mAU*s]	Height [mAU]	Area %
1	23.928	BB	0.5732	382.14053	9.54243	16.4998
2	31.530	BB	0.8187	1933.88562	34.88565	83.5002

Totals :	2316.02615	44.42808

Figure S19. KCl (Table 1 Entry 9).

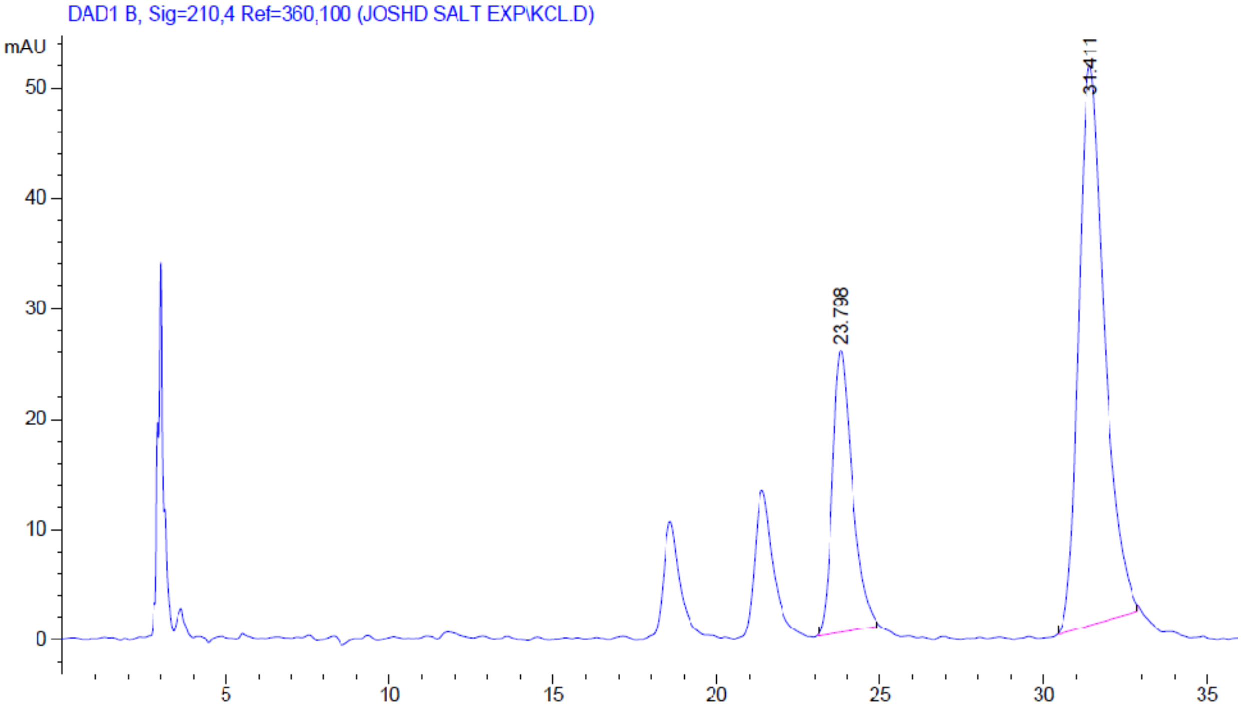

Signal 1: DAD1 B, Sig=210,4 Ref=360,100

Peak #	RetTime [min]	Type	Width [min]	Area [mAU*s]	Height [mAU]	Area %
1	23.798	BB	0.6253	1070.01941	25.51163	27.6610
2	31.411	BB	0.8233	2798.31812	50.74677	72.3390
Totals :				3868.33752	76.25839	

Figure S20. KBr (Table 1. Entry 10).

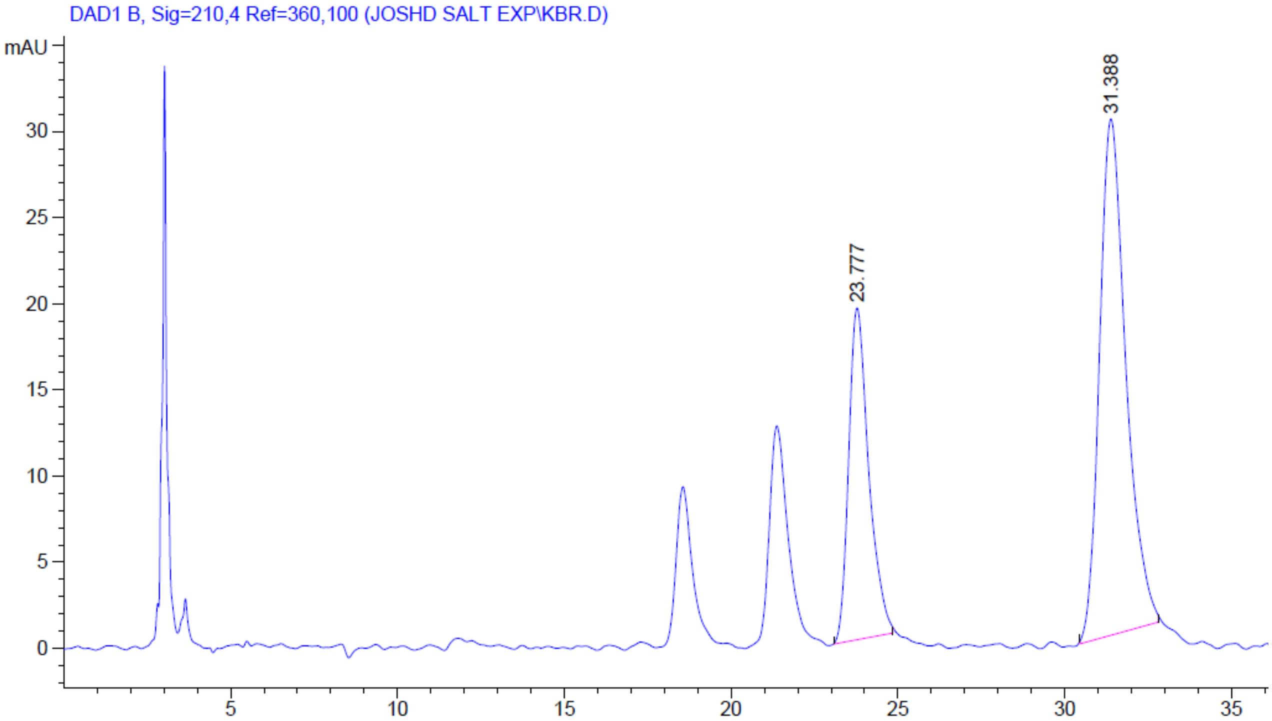

Signal 1: DAD1 B, Sig=210,4 Ref=360,100

Peak #	RetTime [min]	Type	Width [min]	Area [mAU*s]	Height [mAU]	Area %
1	23.777	BB	0.6168	803.50037	19.25532	32.7264
2	31.388	BB	0.8150	1651.70789	29.97088	67.2736

Totals : 2455.20825 49.22619

Figure S21. KI (Table 1. Entry 11).

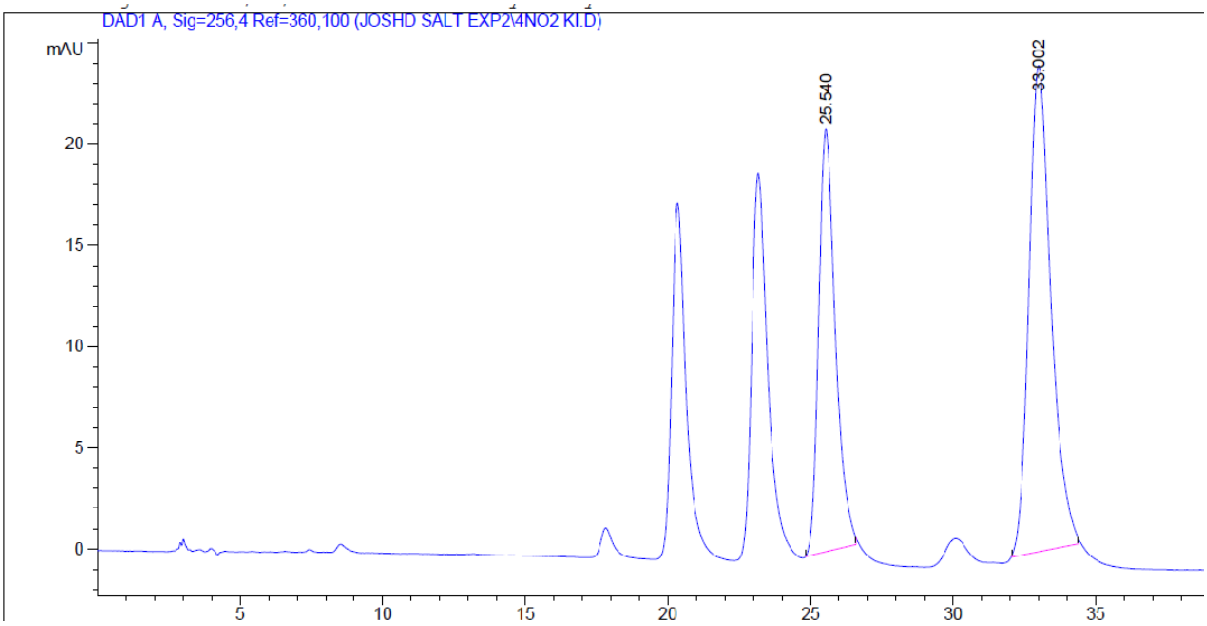

Signal 1: DAD1 A, Sig=256,4 Ref=360,100

Peak #	RetTime [min]	Type	Width [min]	Area [mAU*s]	Height [mAU]	Area %
1	25.540	BB	0.6069	849.36493	20.86427	39.5998
2	33.002	BB	0.8153	1295.50867	24.01902	60.4002

Totals : 2144.87360 44.88329

Figure S22. $MgCl_2$ (Table 1. Entry 12).

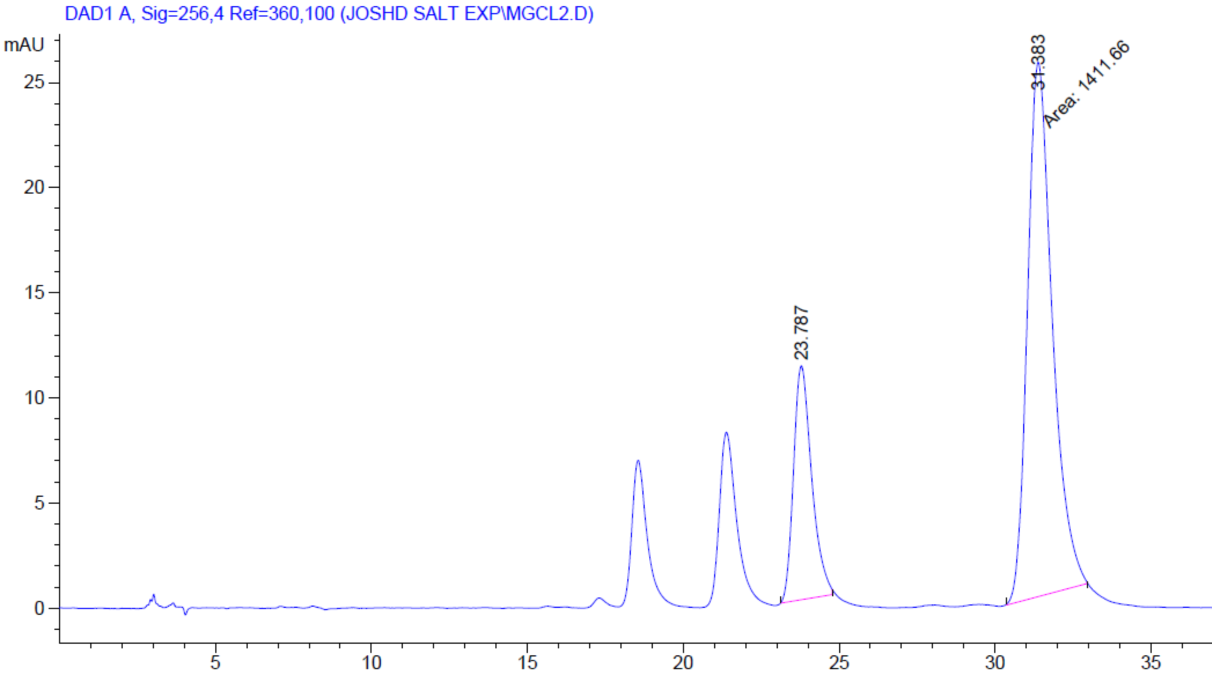

Signal 1: DAD1 A, Sig=256,4 Ref=360,100

Peak #	RetTime [min]	Type	Width [min]	Area [mAU*s]	Height [mAU]	Area %
1	23.787	BB	0.6070	451.06885	11.12597	24.2155
2	31.383	MM	0.9254	1411.65613	25.42332	75.7845

Totals : 1862.72498 36.54929

Figure S23. $CaCl_2$ (Table 1. Entry 13).

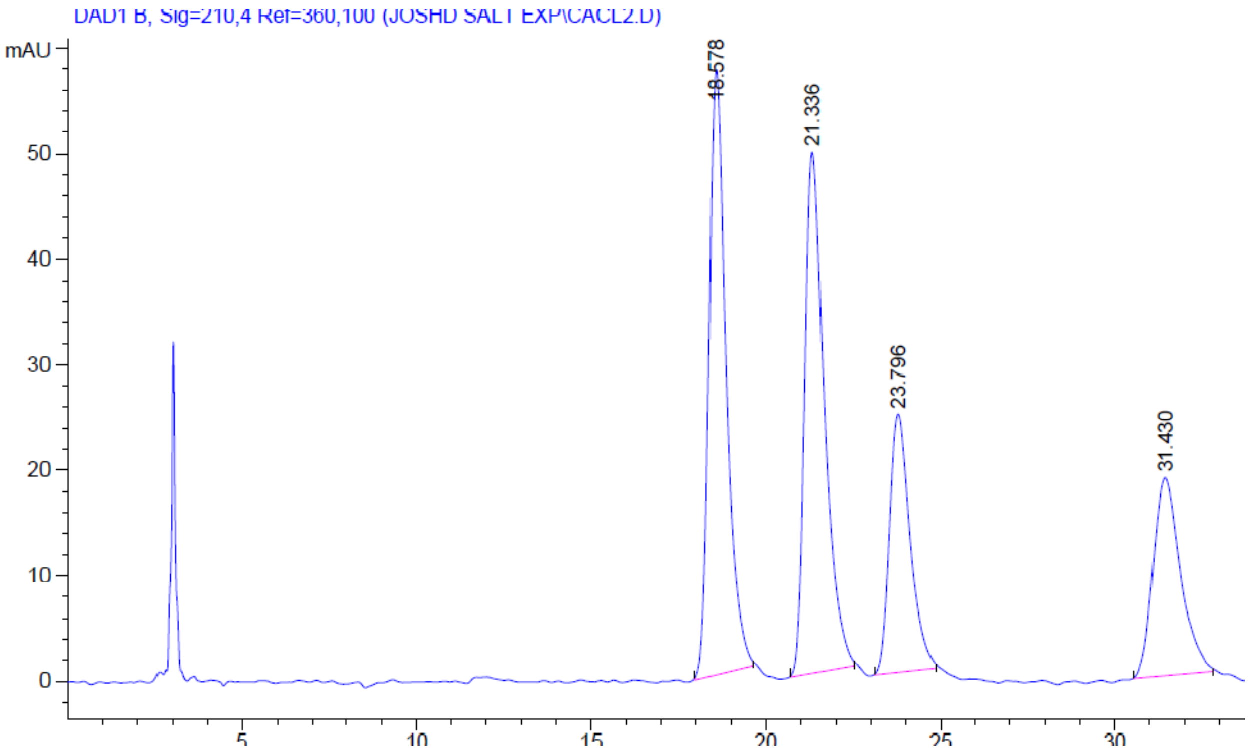

Signal 1: DAD1 B, Sig=210,4 Ref=360,100

Peak #	RetTime [min]	Type	Width [min]	Area [mAU*s]	Height [mAU]	Area %
1	18.578	BB	0.5044	1929.41626	57.35960	32.9100
2	21.336	BB	0.5725	1900.60657	49.43597	32.4186
3	23.796	BB	0.6216	1013.38007	24.54660	17.2852
4	31.430	BB	0.7955	1019.29962	18.77133	17.3862

Totals :	5862.70251	150.11350

Figure S24. $FeCl_3$ (Table 2. Entry 3).

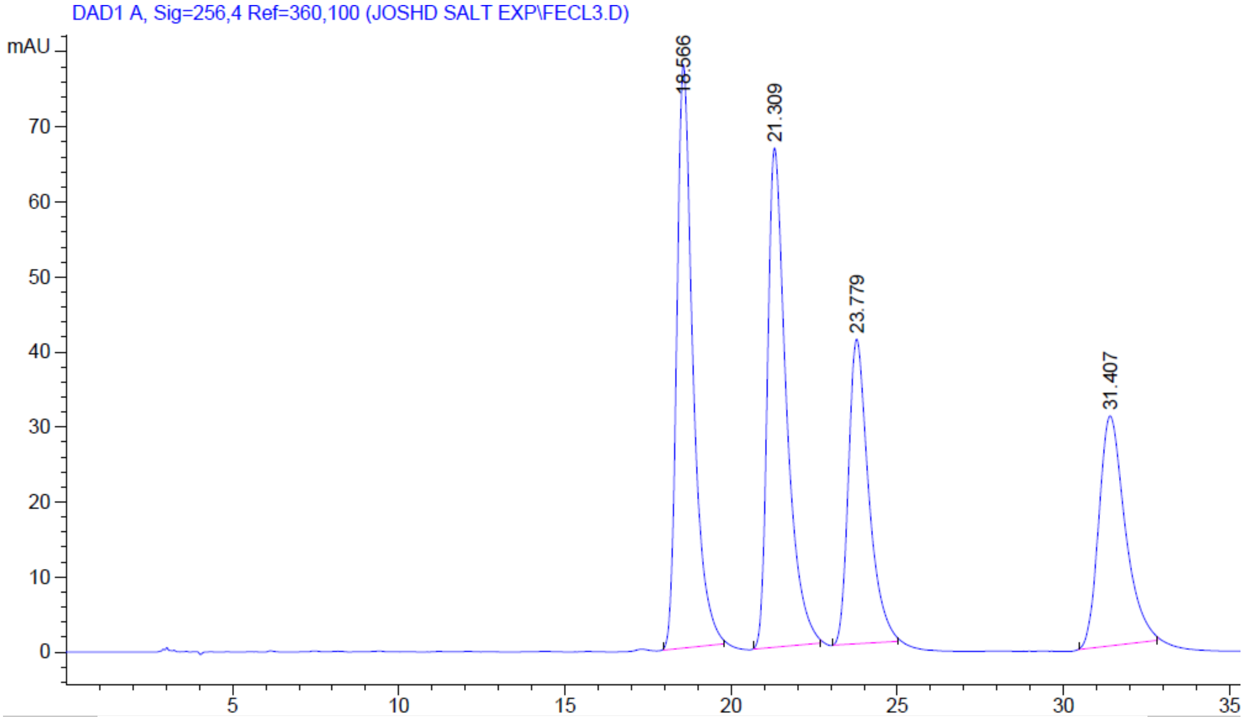

Signal 1: DAD1 A, Sig=256,4 Ref=360,100

Peak #	RetTime [min]	Type	Width [min]	Area [mAU*s]	Height [mAU]	Area %
1	18.566	BB	0.5051	2646.53809	77.74623	30.5194
2	21.309	BB	0.5880	2624.30933	66.55499	30.2631
3	23.779	BB	0.6322	1712.61536	40.59334	19.7496
4	31.407	BB	0.8247	1688.18457	30.64598	19.4679
Totals :				8671.64734	215.54055	

Figure S25. $NiCl_2$ (Table 2. Entry 4).

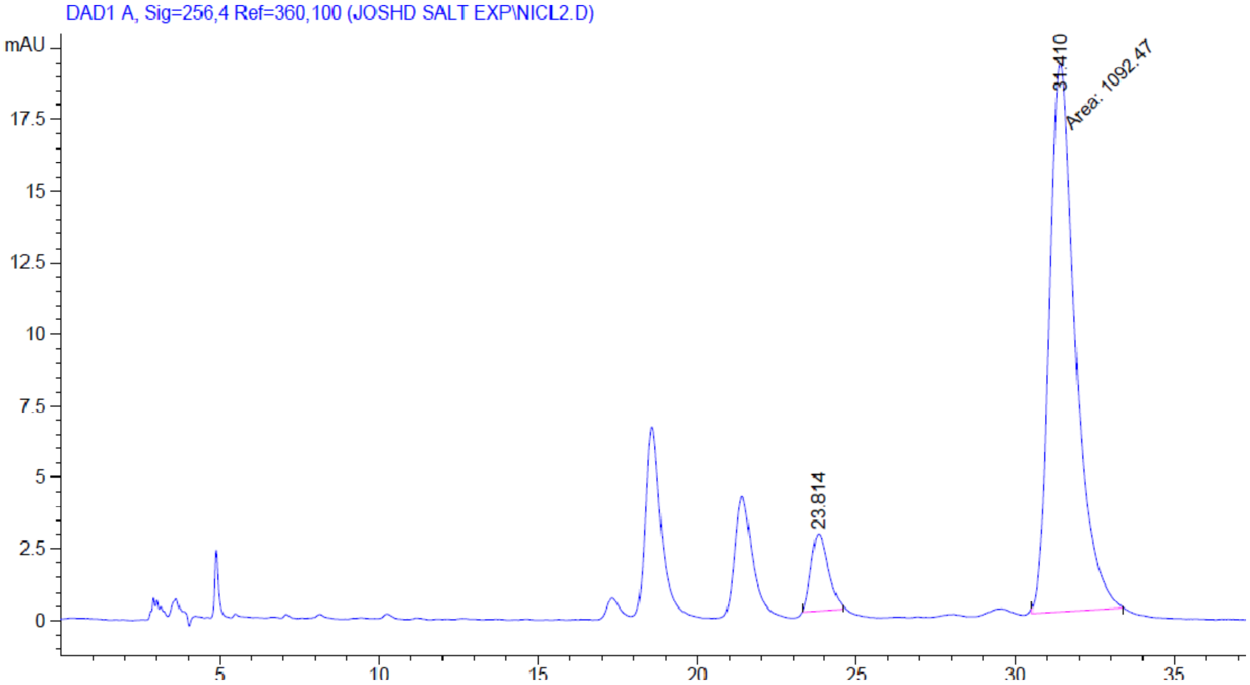

Signal 1: DAD1 A, Sig=256,4 Ref=360,100

Peak #	RetTime [min]	Type	Width [min]	Area [mAU*s]	Height [mAU]	Area %
1	23.814	BB	0.5484	100.19231	2.67867	8.4007
2	31.410	MM	0.9487	1092.46948	19.19181	91.5993
Totals :				1192.66179	21.87049	

Figure S26. ZnOAc (Table 2. Entry 5).

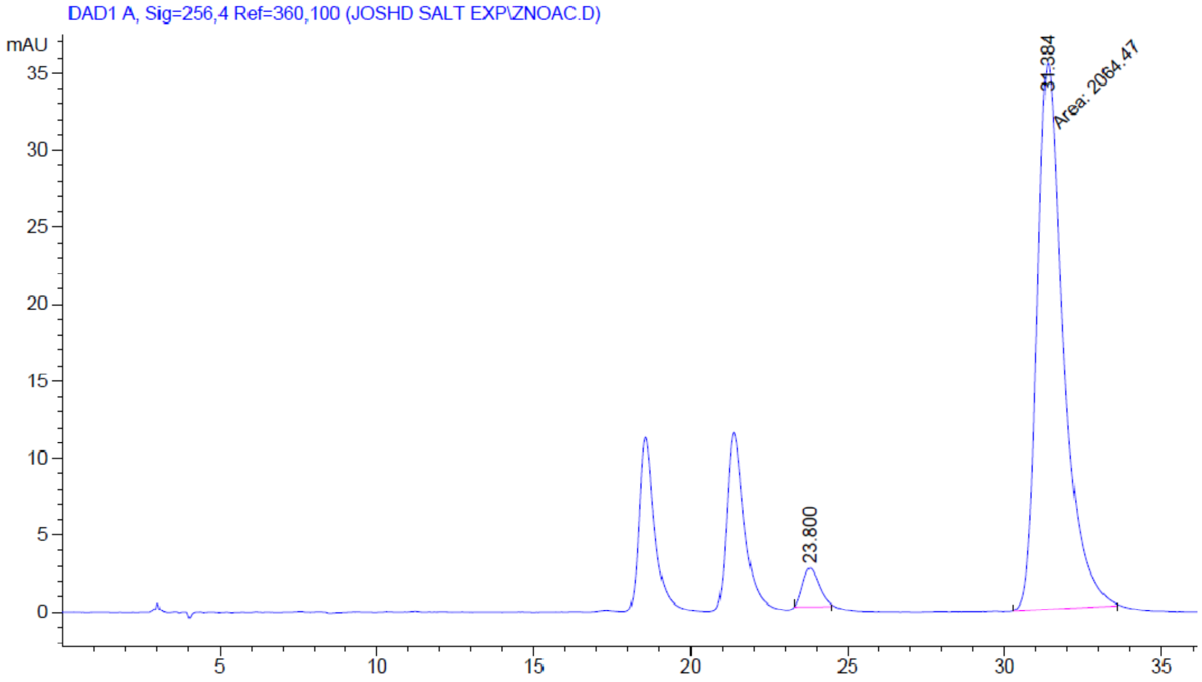

Signal 1: DAD1 A, Sig=256,4 Ref=360,100

Peak #	RetTime [min]	Type	Width [min]	Area [mAU*s]	Height [mAU]	Area %
1	23.800	BB	0.5215	90.98940	2.50675	4.2213
2	31.384	MM	0.9698	2064.47485	35.47806	95.7787

Totals :				2155.46425	37.98481	

Figure S27. EDTA in tap water (5% w/w) (Table 3. Entry 3).

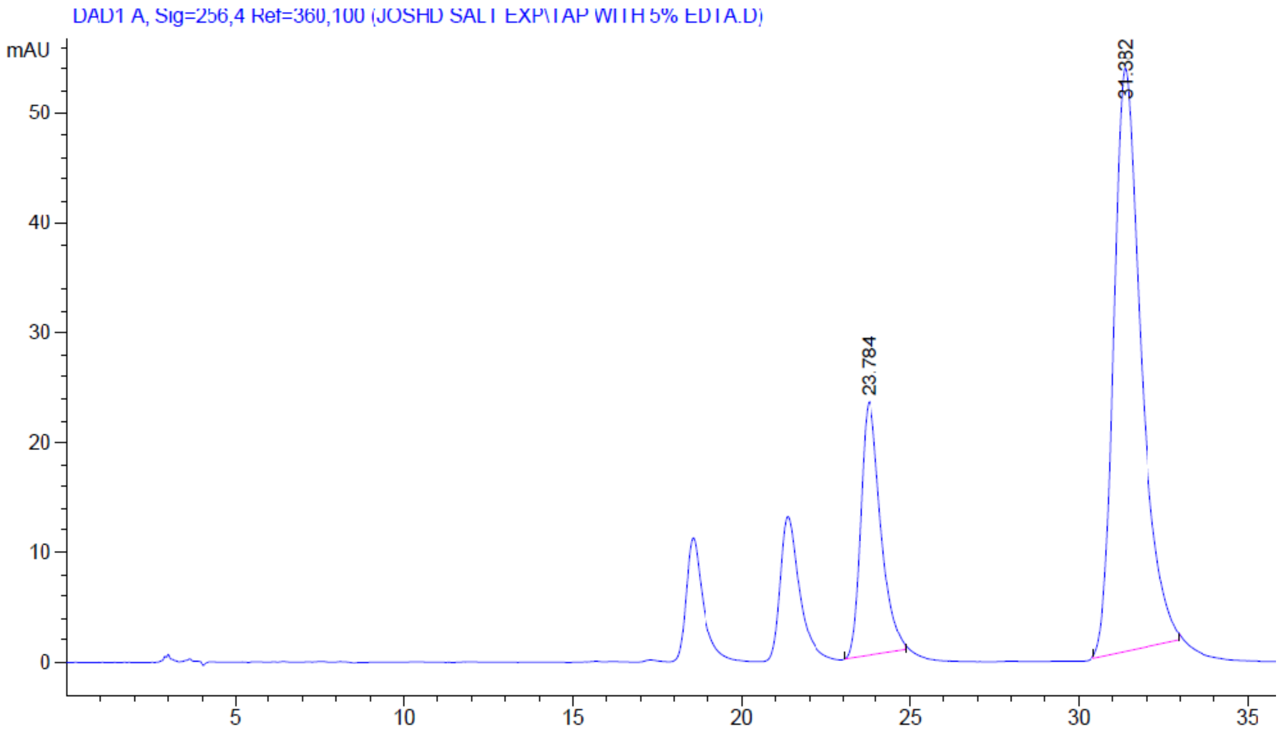

Signal 1: DAD1 A, Sig=256,4 Ref=360,100

Peak #	RetTime [min]	Type	Width [min]	Area [mAU*s]	Height [mAU]	Area %
1	23.784	BB	0.6181	954.94098	23.10849	24.3120
2	31.382	BB	0.8352	2972.91479	53.09694	75.6880
Totals :				3927.85577	76.20543	

Figure S28. 2-[Hydroxy-(phenyl)-methyl]-cyclohexanone.

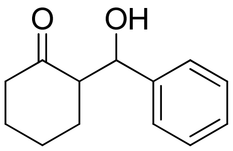

Racemate

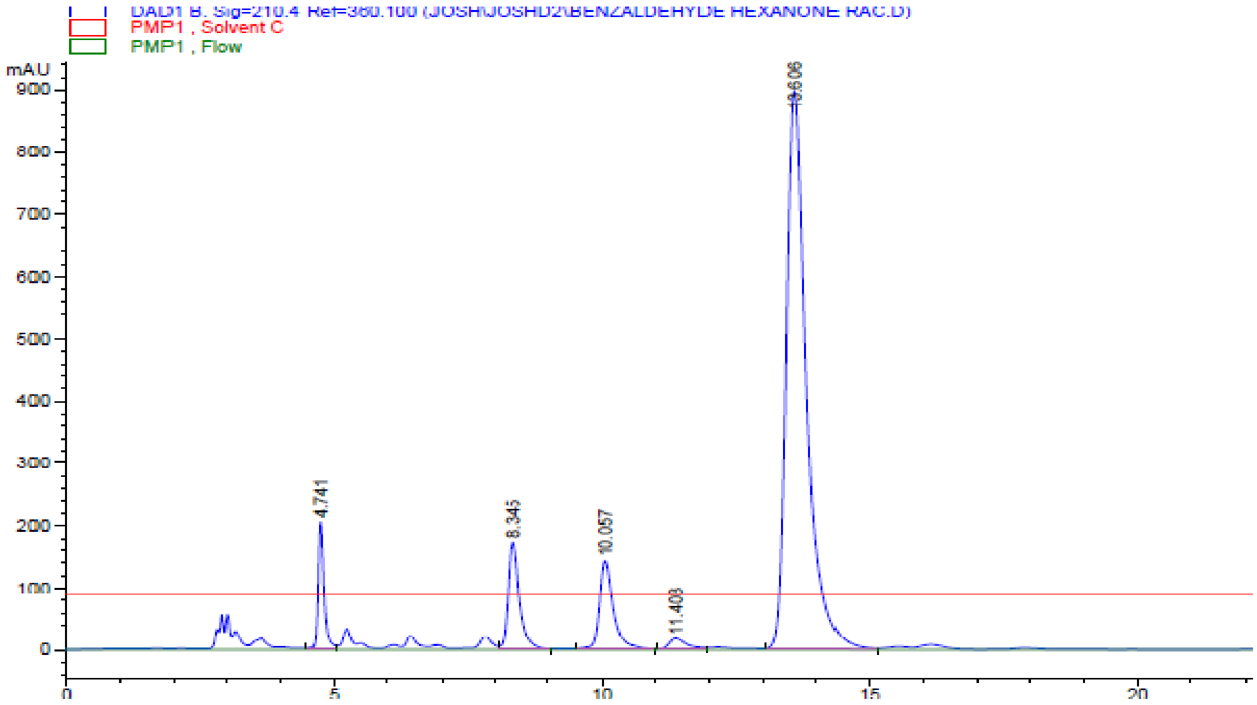

Signal 1: DAD1 B, Sig=210,4 Ref=360,100

Peak #	RetTime [min]	Type	Width [min]	Area [mAU*s]	Height [mAU]	Area %
1	4.741	VV	0.1172	1598.28735	204.39705	4.7671
2	8.345	VB	0.2121	2479.68359	172.13239	7.3960
3	10.057	BB	0.2677	2567.86621	141.48685	7.6590
4	11.403	BB	0.3225	373.11096	16.97196	1.1128
5	13.606	BB	0.3943	2.37050e4	896.97095	70.7031
6	30.788	BB	0.8742	2803.60693	43.22908	8.3621

Totals : 3.35276e4 1480.18826

Figure S29. DI water (Table 4. Entry 1).

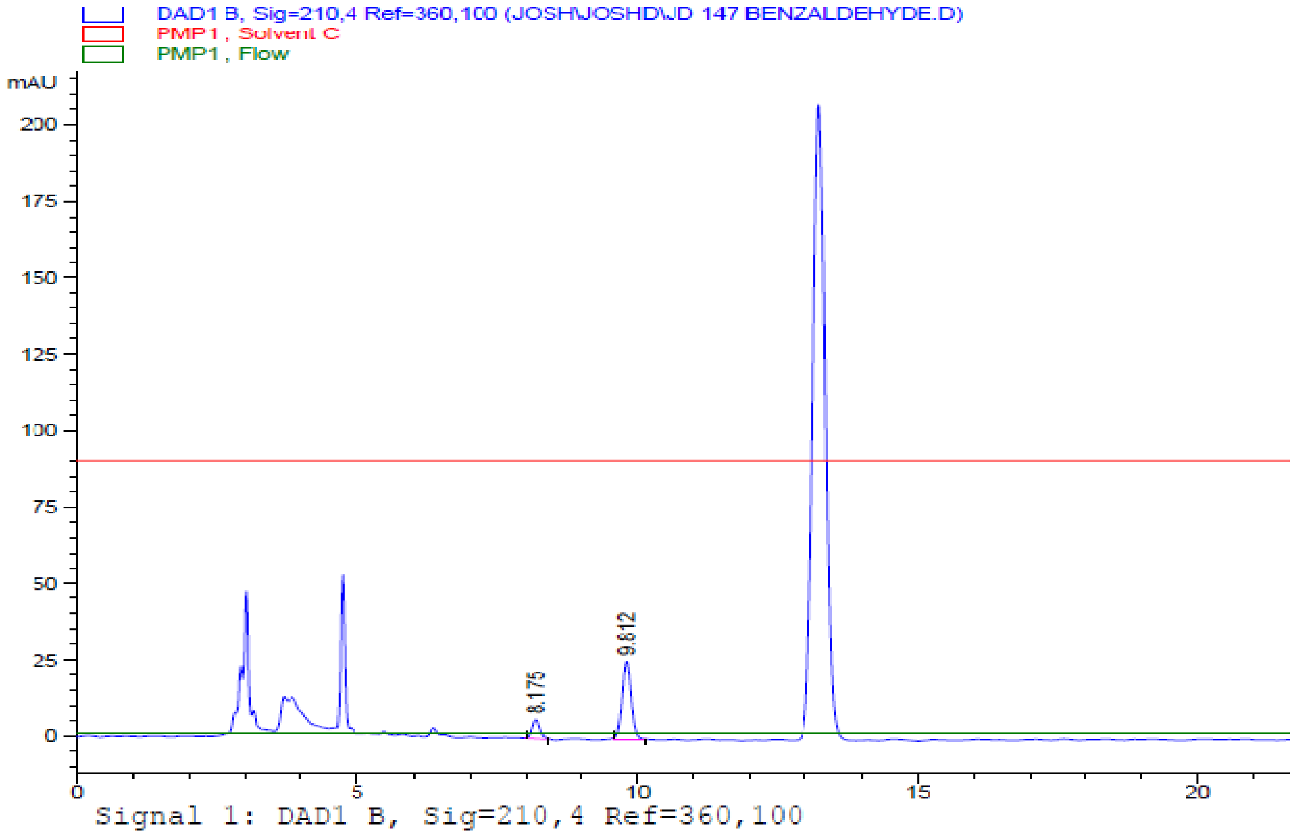

Signal 1: DAD1 B, Sig=210,4 Ref=360,100

Peak #	RetTime [min]	Type	Width [min]	Area [mAU*s]	Height [mAU]	Area %
1	8.175	BB	0.1476	59.21001	6.18892	17.0732
2	9.812	BB	0.1730	287.59024	25.61430	82.9268
Totals :				346.80025	31.80321	

Figure S30. Tap water (Table 4. Entry 2).

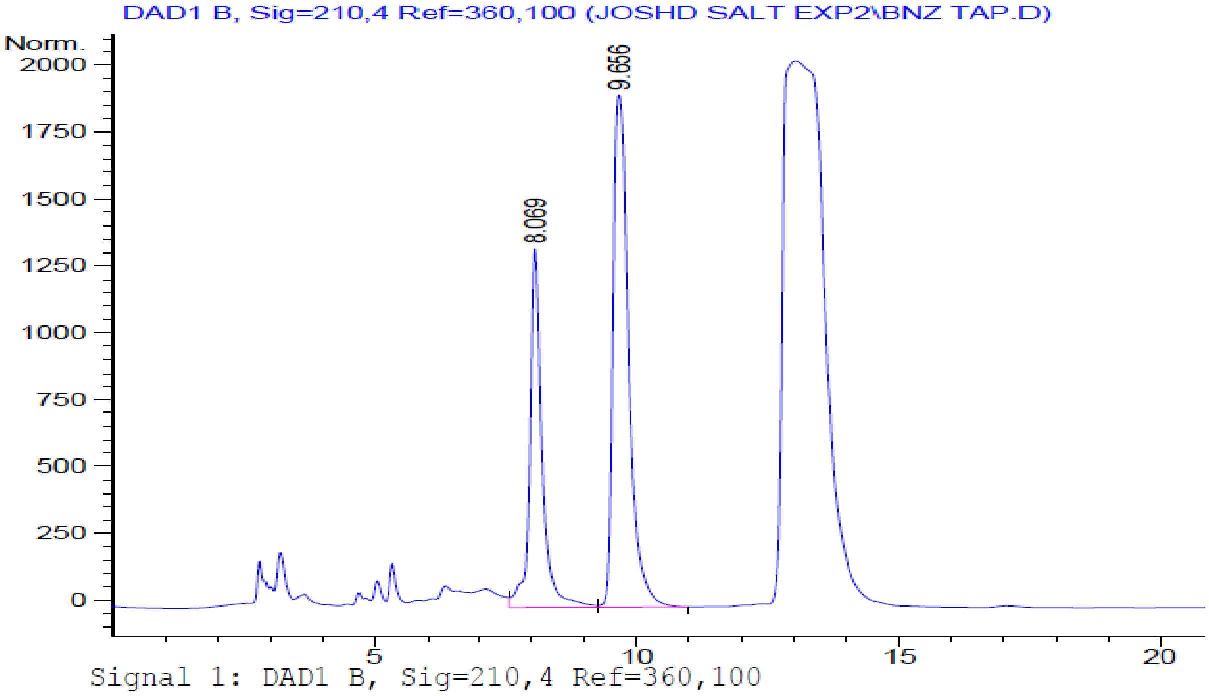

Signal 1: DAD1 B, Sig=210,4 Ref=360,100

Peak #	RetTime [min]	Type	Width [min]	Area [mAU*s]	Height [mAU]	Area %
1	8.069	VV	0.2416	2.22690e4	1342.13904	35.8775
2	9.656	VB	0.3162	3.98006e4	1917.19153	64.1225

Totals : 6.20696e4 3259.33057

Figure S31. EDTA in tap water (5% w/w) (Table 4. Entry 3)

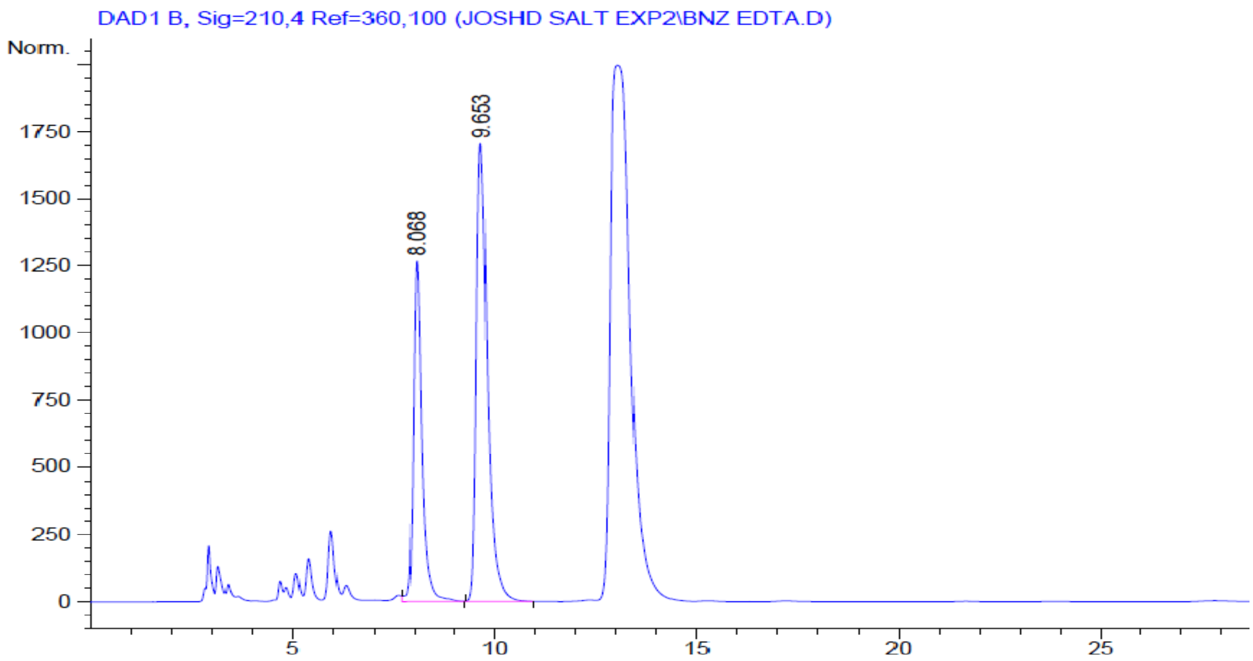

Signal 1: DAD1 B, Sig=210,4 Ref=360,100

Peak #	RetTime [min]	Type	Width [min]	Area [mAU*s]	Height [mAU]	Area %
1	8.069	VV	0.2416	2.22690e4	1342.13904	35.8775
2	9.656	VB	0.3162	3.98006e4	1917.19153	64.1225

Totals : 6.20696e4 3259.33057

Figure S32. $ZnOAc_2$ (Table 4, Entry 4).

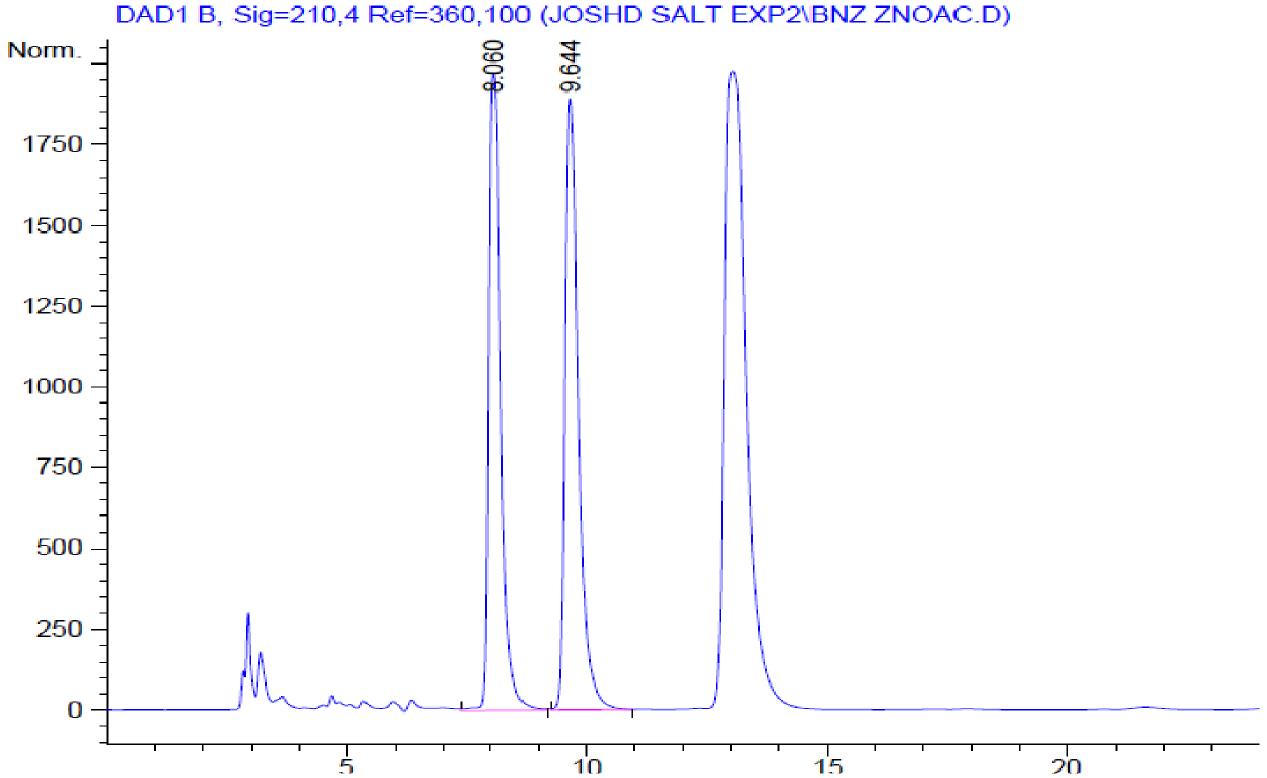

Signal 1: DAD1 B, Sig=210,4 Ref=360,100

Peak #	RetTime [min]	Type	Width [min]	Area [mAU*s]	Height [mAU]	Area %
1	8.060	VB	0.2742	3.51967e4	1972.23157	48.0128
2	9.644	BB	0.3069	3.81103e4	1892.85742	51.9872

Totals : 7.33070e4 3865.08899

Figure S33. 2-[Hydroxy-(4-fluoro-phenyl)-methyl]-cyclohexanone.

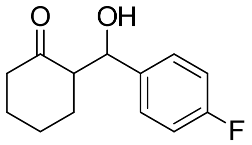

Racemate

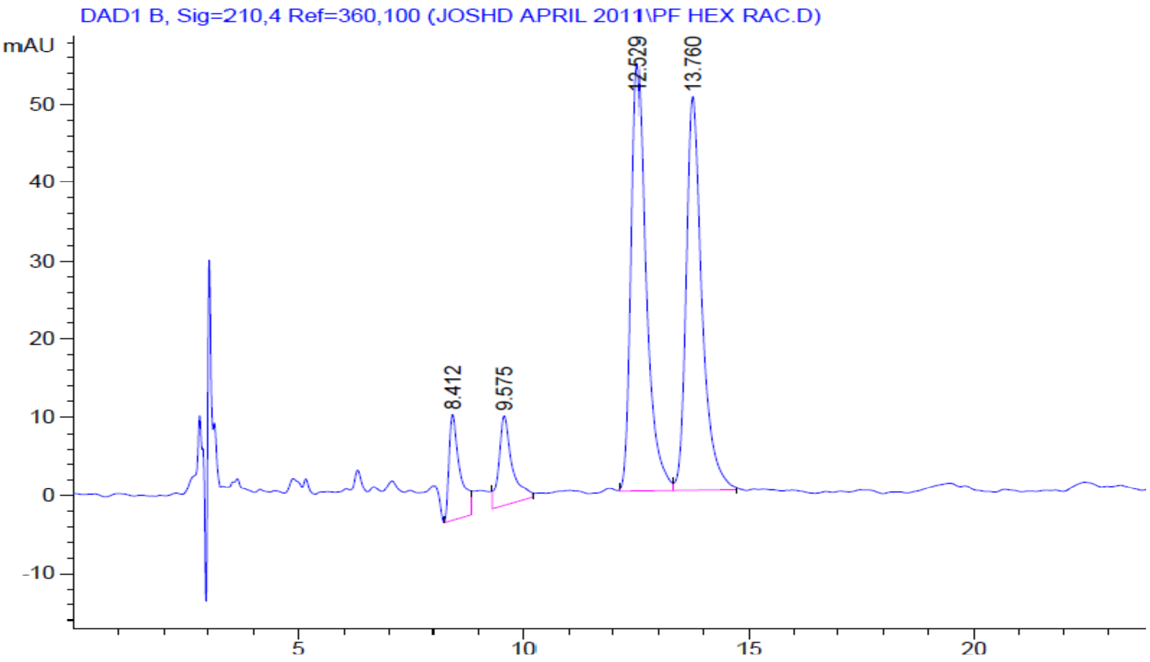

Signal 1: DAD1 B, Sig=210,4 Ref=360,100

Peak #	RetTime [min]	Type	Width [min]	Area [mAU*s]	Height [mAU]	Area %
1	8.412	VB	0.2505	233.07262	13.42521	7.9207
2	9.575	BB	0.3017	239.56458	11.37315	8.1413
3	12.529	BV	0.3286	1220.61682	54.63713	41.4810
4	13.760	VB	0.3695	1249.33508	50.33954	42.4570

Figure S34. DI water (Table 4, Entry 5).

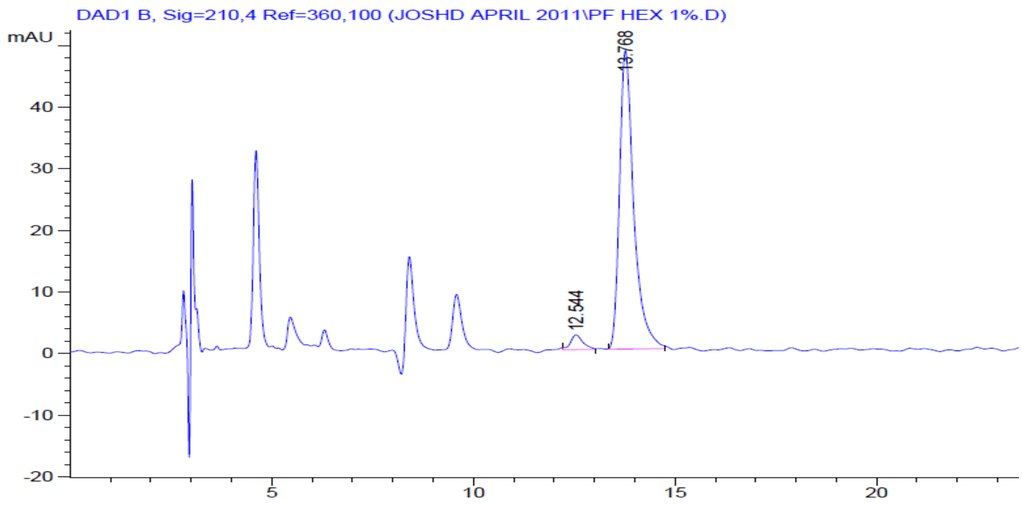

Signal 1: DAD1 B, Sig=210,4 Ref=360,100

Peak #	RetTime [min]	Type	Width [min]	Area [mAU*s]	Height [mAU]	Area %
1	12.544	BB	0.3141	50.61238	2.39917	4.0699
2	13.768	BB	0.3672	1192.96582	48.45242	95.9301

Totals : 1243.57821 50.85159

Figure S35. Tap water (Table 4. Entry 6).

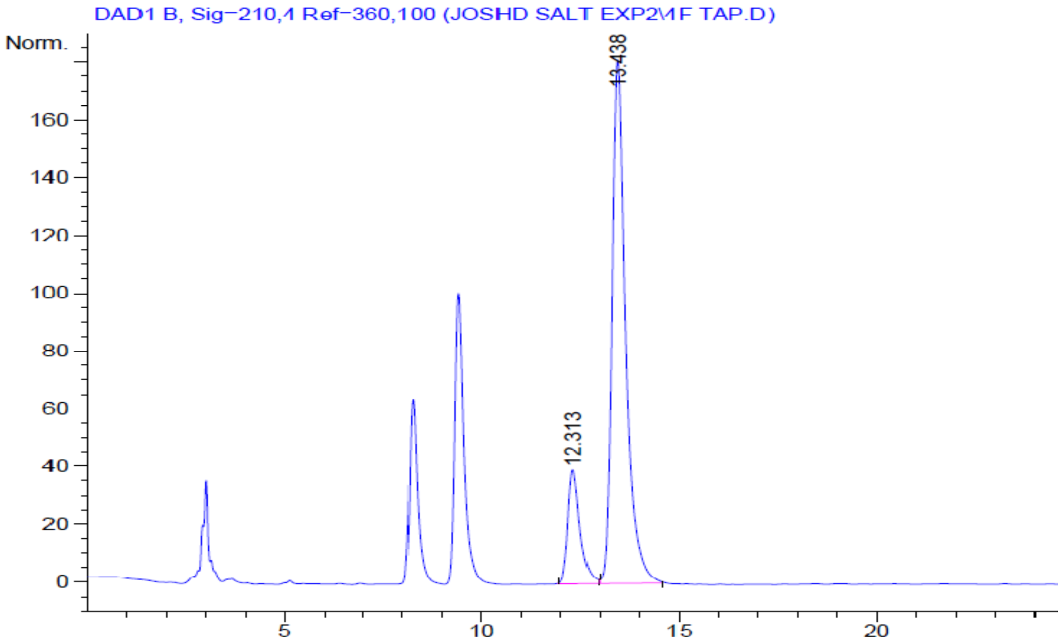

Signal 1: DAD1 B, Sig=210,4 Ref=360,100

Peak #	RetTime [min]	Type	Width [min]	Area [mAU*s]	Height [mAU]	Area %
1	12.313	BB	0.3221	849.05157	39.29692	16.1941
2	13.438	BB	0.3591	4393.92041	181.08508	83.8059

Totals : 5242.97198 220.38200

Figure S36. EDTA in tap water (5% w/w) (Table 4. Entry 7).

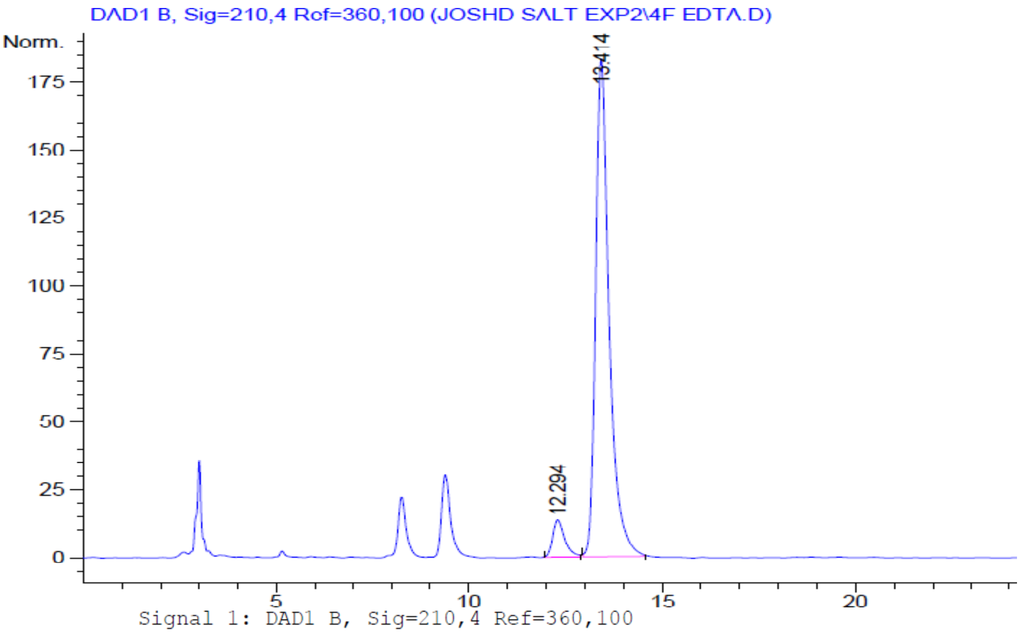

Signal 1: DAD1 B, Sig=210,4 Ref=360,100

Peak #	RetTime [min]	Type	Width [min]	Area [mAU*s]	Height [mAU]	Area %
1	12.294	BB	0.3195	288.20819	13.47921	6.1085
2	13.414	BB	0.3603	4429.94775	183.10228	93.8915
Totals :				4718.15594	196.58149	

Figure S37. $ZnOAc_2$ (Table 4, Entry 8).

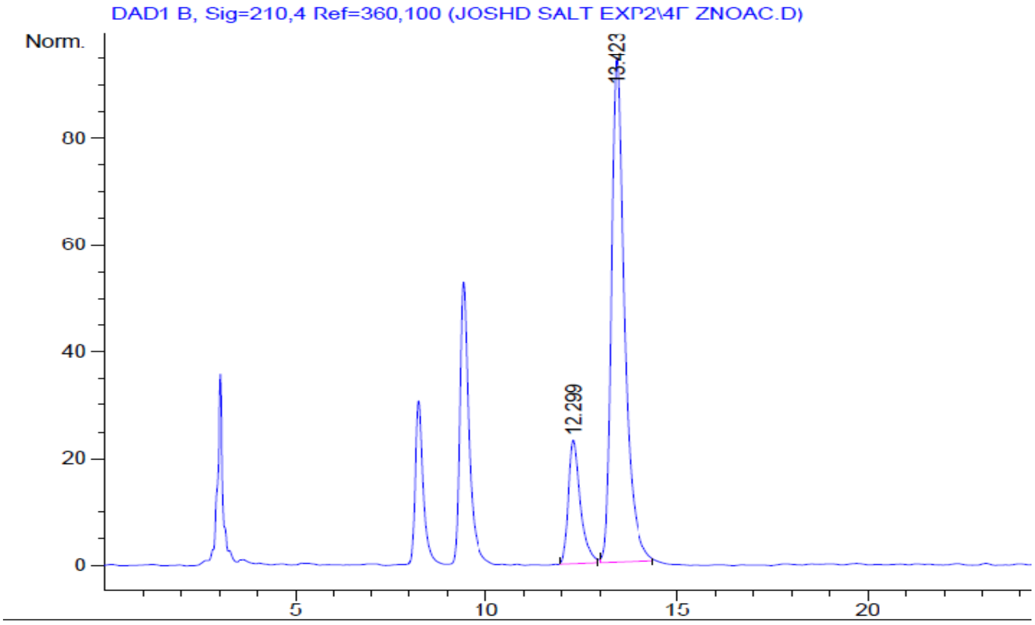

Signal 1: DAD1 B, Sig=210,4 Ref=360,100

Peak #	RetTime [min]	Type	Width [min]	Area [mAU*s]	Height [mAU]	Area %
1	12.299	BB	0.3184	492.06708	23.10807	17.9518
2	13.423	BB	0.3541	2248.98193	94.33146	82.0482
Totals :				2741.04901	117.43953	

Figure S38. 2-[Hydroxy-(4-nitro-phenyl)-methyl]-cyclopentanone.

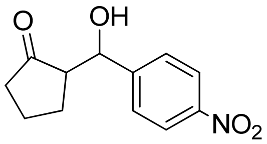

Racemate

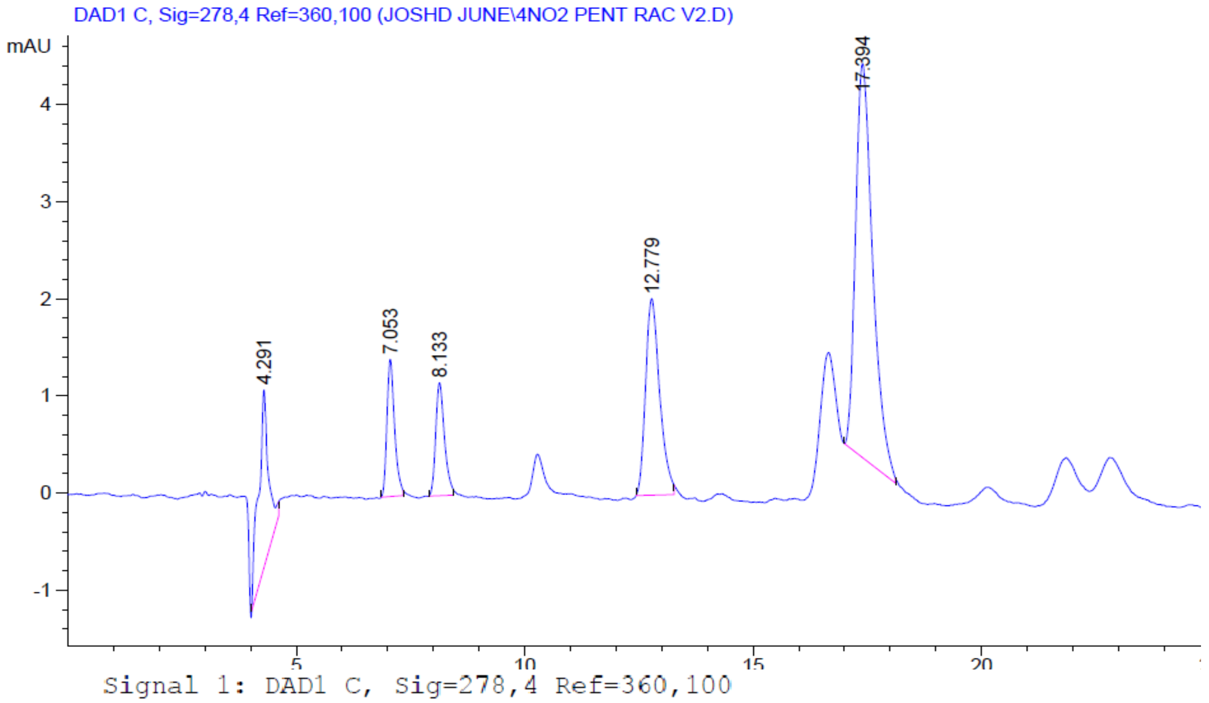

Signal 1: DAD1 C, Sig=278,4 Ref=360,100

Peak #	RetTime [min]	Type	Width [min]	Area [mAU*s]	Height [mAU]	Area %
1	4.291	BB	0.2023	27.93900	1.82841	13.3428
2	7.053	BB	0.1733	16.38140	1.41283	7.8232
3	8.133	BB	0.2005	15.59259	1.16262	7.4465
4	12.779	BB	0.3093	43.58838	2.02334	20.8164
5	17.394	BB	0.3886	105.89257	4.05558	50.5710

Figure S39. DI water (Table 4. Entry 9).

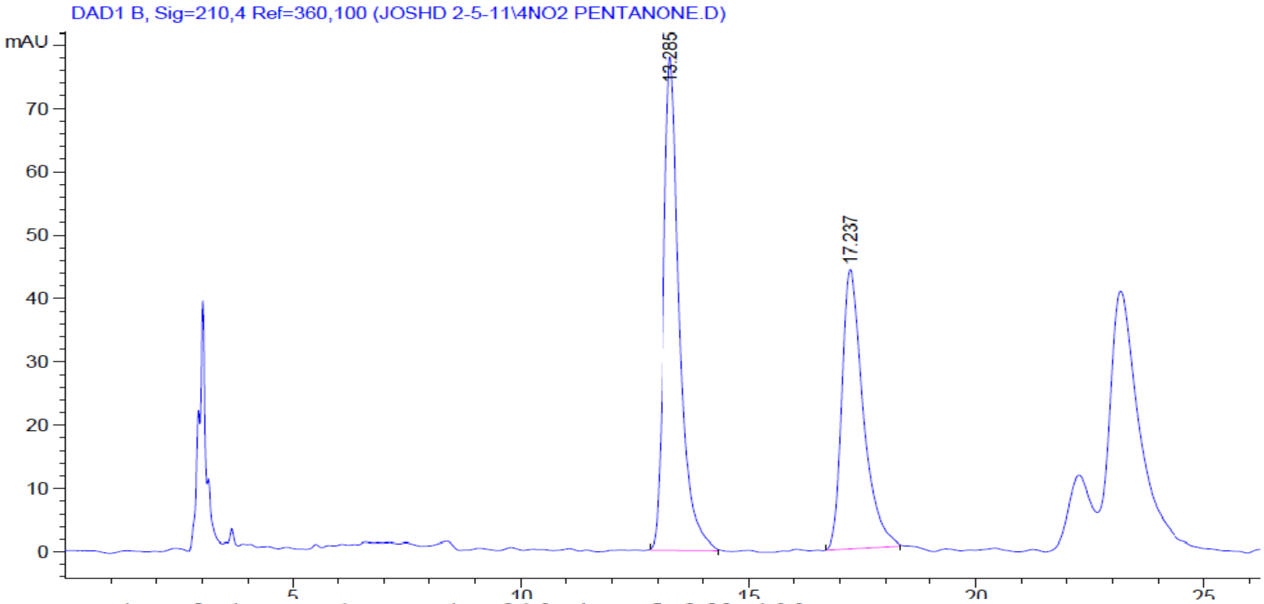

Signal 1: DAD1 B, Sig=210,4 Ref=360,100

Peak #	RetTime [min]	Type	Width [min]	Area [mAU*s]	Height [mAU]	Area %
1	13.285	BB	0.3633	1905.12170	77.91515	57.2065
2	17.237	BB	0.4848	1425.13110	44.13097	42.7935
Totals :				3330.25281	122.04612	

Figure S40. Tap water (Table 4. Entry 10).

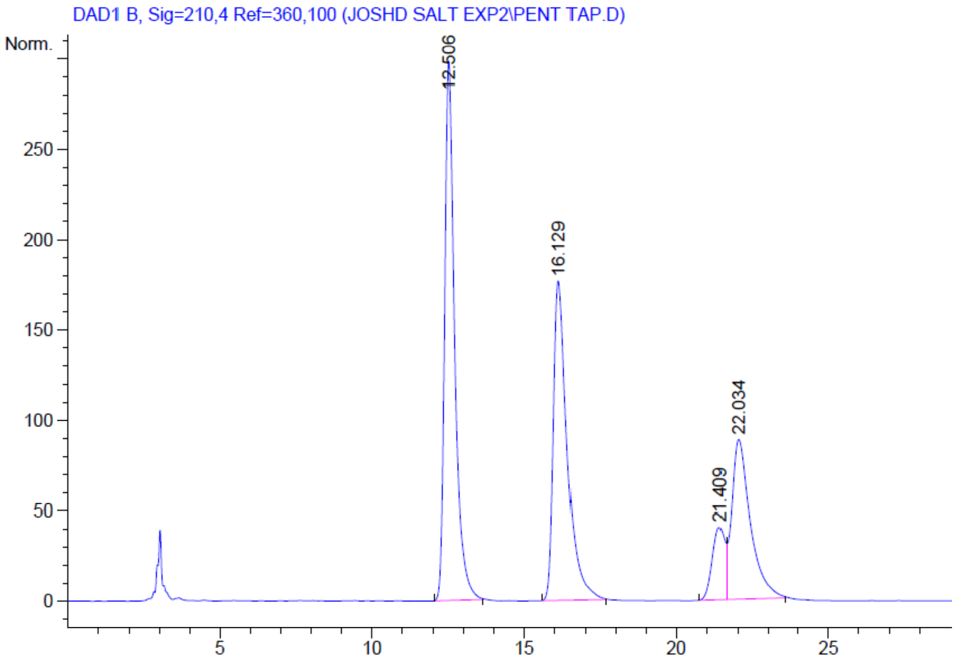

Signal 1: DAD1 B, Sig=210,4 Ref=360,100

Peak #	RetTime [min]	Type	Width [min]	Area [mAU*s]	Height [mAU]	Area %
1	12.506	BB	0.3456	6888.05859	298.08484	39.6544
2	16.129	BB	0.4629	5562.37500	176.86067	32.0224
3	21.409	BV	0.4301	1125.14148	40.25286	6.4774
4	22.034	VB	0.6186	3794.66211	88.43087	21.8458

Figure S41. EDTA in tap water (5% w/w) (Table 4. Entry 11).

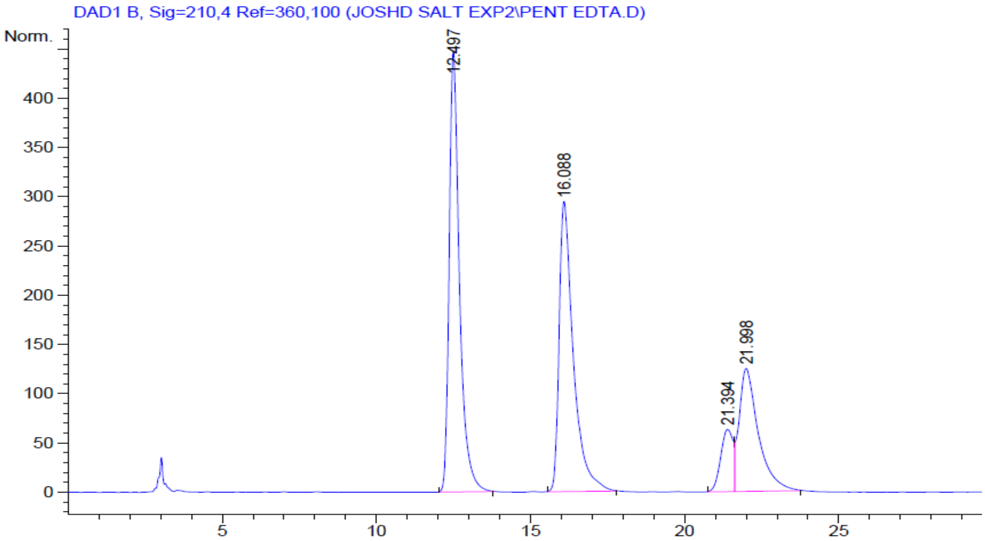

Signal 1: DAD1 B, Sig=210,4 Ref=360,100

Peak #	RetTime [min]	Type	Width [min]	Area [mAU*s]	Height [mAU]	Area %
1	12.497	BB	0.3472	1.04121e4	447.95496	38.5339
2	16.088	BB	0.4686	9411.18945	294.71713	34.8297
3	21.394	BV	0.4257	1752.27869	63.15086	6.4850
4	21.998	VB	0.6291	5445.03809	124.78777	20.1514

Figure S42. ZnOAc (Table 4. Entry 12).

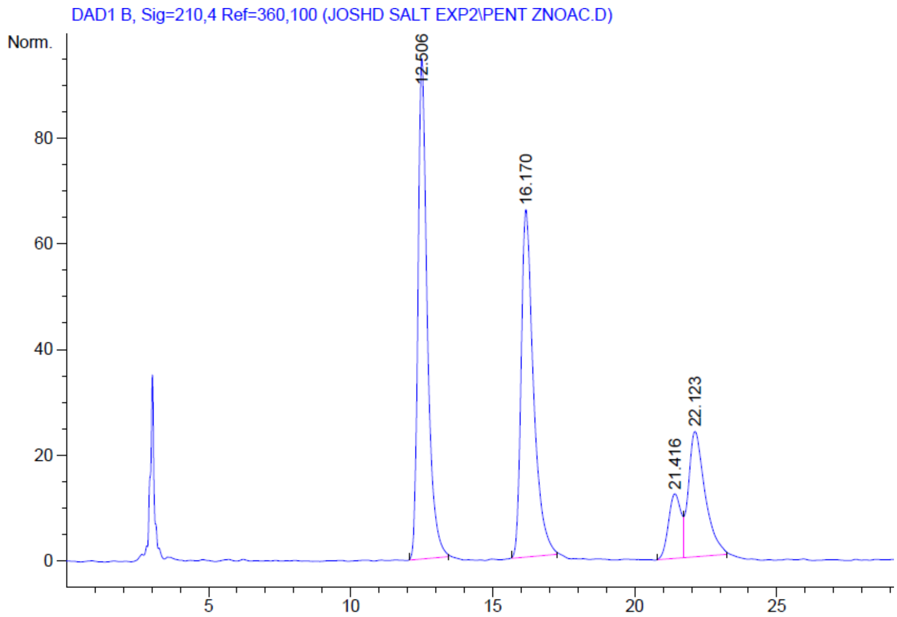

Signal 1: DAD1 B, Sig=210,4 Ref=360,100

Peak #	RetTime [min]	Type	Width [min]	Area [mAU*s]	Height [mAU]	Area %
1	12.506	BB	0.3409	2165.08447	94.64420	39.4235
2	16.170	BB	0.4461	1984.48633	65.74780	36.1350
3	21.416	BV	0.4576	371.73959	12.19679	6.7689
4	22.123	VB	0.6015	970.55792	23.71293	17.6726

Reference

1. Giacalone, F.; Gruttadauria, M.; Meo, P.L.; Reila, S.; Noto, R. New Simple Hydrophobic Proline Derivatives as Highly Active and Stereoselective Catalysts for the Direct Asymmetric Aldol Reaction in Aqueous Medium. *Adv. Synth. Catal.* **2008**, *350*, 2747–2760.

© 2011 by the authors; licensee MDPI, Basel, Switzerland. This article is an open access article distributed under the terms and conditions of the Creative Commons Attribution license (http://creativecommons.org/licenses/by/3.0/).
